# Cancer Cell CD73 Loss Promotes a Dual Inflammatory and Immunosuppressive Microenvironment in Endometrial Carcinoma

**DOI:** 10.1158/2767-9764.CRC-26-0167

**Published:** 2026-11-05

**Authors:** Hannah N. Lee, Xingyuan Zhang, Suzanne M. Crumley, Emily M. Rabjohns, Luan H. Phan, Di Wu, Russell R. Broaddus, Cyrus Vaziri, Jessica L. Bowser

**Affiliations:** 1 Department of Pathology and Laboratory Medicine, University of North Carolina School of Medicine, Chapel Hill, North Carolina.; 2 Department of Pathology and Immunology, Washington University School of Medicine in St. Louis, St. Louis, Missouri.; 3 Graduate Curriculum in Pathobiology and Translational Science, University of North Carolina at Chapel Hill, Chapel Hill, North Carolina.; 4 Department of Internal Medicine, University of Texas McGovern Medical School, Houston, Texas.; 5 Department of Biostatistics, Gillings School of Global Public Health, University of North Carolina at Chapel Hill, Chapel Hill, North Carolina.; 6 Department of Biomedical Sciences, Adams School of Dentistry, University of North Carolina at Chapel Hill, Chapel Hill, North Carolina.; 7 UNC Lineberger Comprehensive Cancer Center, University of North Carolina at Chapel Hill, Chapel Hill, North Carolina.

## Abstract

CD73 is an emerging immunotherapy target that is overexpressed in many tumors and generates an adenosine-rich microenvironment to suppress antitumor immunity. In low-grade endometrial carcinoma, CD73 expression varies, and its impact on tumor immunobiology is unknown. Using two microsatellite-stable, grade 2 endometrioid endometrial carcinoma cohorts, *in vitro NT5E* (CD73) knockout/knockdown, and single-cell and spatial analyses, we show that cancer cell CD73 loss promotes a dual inflammatory and immunosuppressive microenvironment. Cancer cell CD73 loss was associated with increased lymphovascular space invasion and worse recurrence-free and overall survival, whereas stromal CD73 expression did not correlate with outcomes. CD73 downregulation induced pro-inflammatory gene expression, which strongly correlated with upregulation of immunosuppressive genes, including *CD274* (PD-L1), suggesting a compensatory mechanism limiting antitumor immunity. Tumors with low cancer cell CD73 exhibited higher PD-L1 expression that correlated with increased CD3^+^ T-cell infiltration, reflecting an immune-engaged but checkpoint-restrained microenvironment. Single-cell analyses demonstrated that *NT5E+* nonimmune cells in immune-responsive clusters were three times less likely to express *CD274*. In clinically relevant low-grade endometrial carcinoma models, functional studies confirmed that CD73 loss promoted both pro-inflammatory and immunosuppressive programs. These results reveal a context-specific role for CD73 in shaping the immune landscape of low-grade endometrial carcinoma that is distinct from its function in other solid tumors.

**Significance:**

Unlike most cancers, in which CD73 drives immunosuppression via overexpression, we show that cancer cell CD73 loss promotes an inflammatory yet compensatory immunosuppressive microenvironment in low-grade endometrial carcinoma, revealing a context-specific role. CD73 loss in endometrial carcinoma therefore may be as immunologically significant as its overexpression in other cancers.

## Introduction

CD73, a cell surface 5′ nucleotidase that generates extracellular adenosine, is a well-established mediator of immunosuppression in cancer. Extracellular adenosine is immunosuppressive in both normal and cancerous tissues, and many tumors upregulate CD73 to evade antitumor immune cell responses (1, 2). Small-molecule inhibitors and antibodies that inhibit CD73 are currently being evaluated in clinical trials, often in combination with immune checkpoint blockade or chemotherapy (1, 2). CD73 expression differs in endometrial carcinomas. Rather than being overexpressed, CD73 is frequently downregulated in high-grade endometrial tumors, and its loss is associated with prognostically poor clinical features and worse survival outcomes (3). Similar associations between CD73 loss and adverse clinical features or poor prognosis have been reported in prostate and bladder cancers (4, 5). Although tumor-promoting functions of CD73 are well-understood, why some tumors downregulate CD73 remains largely unknown. As targeted therapy for CD73 continues to advance, understanding CD73–adenosine biology in cancers with more diverse expression will be critical for personalized treatment strategies.

Increasing evidence suggests that immune evasion is important not only to high-grade endometrial carcinomas but also to disease progression in low-grade tumors (6–8). Low-grade (grade 1 and 2) endometrioid endometrial carcinomas (EEC) are the most common endometrial carcinoma subtype. Although most patients present with early-stage disease and favorable prognosis, approximately 5% to 10% experience recurrence and poor survival outcomes (6). One possible explanation for the aggressiveness of these tumors is the gain of mechanisms that promote immunosuppression (6, 7). Although CD73 is downregulated in high-grade EECs and endometrial serous carcinomas, CD73 expression in low-grade EECs is highly variable, ranging from levels comparable with normal endometrium to near complete loss (3). Conceivably, this variability may reflect different pathobiologies. Low-grade EECs are one of the most diverse groups of endometrial tumors in terms of genetic and molecular features (9–11). Considering the variability of CD73 expression and the strong link of CD73 to immunosuppression in other solid tumors, we sought to determine whether CD73 has a role in the immunobiology of low-grade EECs.

We focused on microsatellite-stable (MSS) grade 2 EECs (G2 EEC^MSS^) to avoid confounding effects of highly immunogenic microsatellite instability (MSI)–high (MSI-H) tumors (12). MSI-H and mismatch repair deficiency generally overlap in endometrial carcinoma, with reported discordance rates of approximately 2% to 8% (13). We analyzed G2 EEC^MSS^ from two independent published cohorts (11, 14) and used publicly available single-cell data from six independent endometrial carcinomas (15) and GeoMx digital spatial profiling (DSP) data from low-grade EECs known to be clinically aggressive (9, 16). In elucidating CD73 in the immunobiology of G2 EEC^MSS^, we uncovered that cancer cell CD73 loss promotes a dual inflammatory and immunosuppressive microenvironment in endometrial carcinoma.

## Materials and Methods

### Sex as a biological variable

All samples analyzed were derived from female patients, as endometrial carcinoma occurs exclusively in females.

### Analyses with publicly available datasets

#### The Cancer Genome Atlas-Uterine Corpus Endometrial Carcinoma RNA sequencing analyses

RNA sequencing (RNA-seq) count data from The Cancer Genome Atlas (TCGA) for Uterine Corpus Endometrial Carcinoma (UCEC) were obtained from the Genomic Data Commons portal. Analyses were restricted to G2 EEC^MSS^ tumors, and only tumors in the highest and lowest quartiles of *NT5E* expression were used for downstream analyses. R (version 4.1.0; RRID: SCR_001905) was used for visualizing and data analyses and the R package DESeq2 (version 1.32.0; RRID: SCR_015687) was used for differential expression (DE) analysis. For the DE analysis, genes with log_2_ fold change >1 or < −1 and adjusted *P* value < 0.05 (adjusted by Benjamini–Hochberg adjusted method) in the Wald test of Negative Binomial Generalized Linear Model were considered differentially expressed genes (DEG). Gene Ontology (GO) Enrichment Analysis was performed using the R package clusterProfiler (version 4.8.3; RRID: SCR_016884) using all DEGs. Spearman rank correlation coefficients and Spearman correlation test for DESeq2-normalized gene expression were used as the correlation coefficients and significance when examining the correlation between genes.

#### Single-cell RNA-seq analyses

Publicly available single-cell RNA-seq data from six endometrial tumors were analyzed using Seurat (15).

#### DSP analyses

GeoMx DSP transcriptomic data from nuclear and nonnuclear regions of interest of 16 next-generation sequencing–validated exon 3 *CTNNB1*-mutant EECs were previously described and are publicly available (16). Count data for specific genes were extracted from the original reported analyses (16), provided by R.R. Broaddus.

### Human tissue samples and clinical data

Written informed consent was obtained from all patients in accordance with institutional requirements. The study was approved by The University of Texas MD Anderson Cancer Center Institutional Review Board (LAB01-718) and was conducted in accordance with the ethical principles of the Belmont Report and applicable institutional and federal regulations. Formalin-fixed, paraffin-embedded MSS, mismatch repair–proficient Federation Internationale des Gynaecologistes et Obstetristes (FIGO) grade 2 EECs (denoted as G2 EEC^MSS^) were retrospectively identified from cases collected between 2013 and 2016. Of 132 eligible tumors previously described (14), 119 showed successful CD73 staining and were included in analyses. Tumor grade and histotype were confirmed by hematoxylin and eosin review. Clinical and pathologic data were abstracted from medical records and are summarized in Supplementary Table S1. Next-generation sequencing–confirmed *CTNNB1*-mutant EEC tumors were obtained as previously described (17).

### IHC and whole-slide image scanning

IHC for CD73 was performed using a validated antibody (Cell Signaling Technology, RRID: AB_2716625, cat. #13160) with standardized antigen retrieval and detection. Slides were scanned using an Aperio LVI Real-time Digital Pathology System (Supplementary Fig. S1A and S1B). IHC for CD3, CD8, and PD-L1 was previously performed on the same cohort (14).

### Manual annotation of CD73 staining

CD73 expression was assessed across ≥95% of tumor area and classified separately in cancer and stromal compartments based on subcellular localization and staining intensity (Supplementary Fig. S1A–S1C). Cancer cell patterns were consolidated into three categories: membrane + cytoplasmic staining (normal), downregulated staining, and no staining. Stromal staining was categorized as high (2+), low (1+), or absent. Quantification is reported as percent area of each classifier relative to total annotated area.

### IHC

Slides were deparaffinized and rehydrated, and antigen retrieval was performed (citrate buffer, pH 6) using a Decloaking Chamber (Biocare Medical). Nonspecific binding was blocked using Background Snipper (Biocare Medical). Tissue sections were then incubated with CD73 antibody (Cell Signaling Technology, RRID: AB_2716625, cat. #13160) overnight at 4°C. The universal Dako-Labeled Streptavidin-Biotin2 System, Horseradish Peroxidase (LSAB2) System (Agilent Technologies, RRID: SCR_026027, cat. #K0675), and Dako DAB (Agilent Technologies, RRID: SCR_026028, cat. #K0673) were used to develop the staining, followed by hematoxylin counterstaining (18). Tissue sections were stained in a single batch, with endothelial cell CD73 serving as internal staining controls. Tissue sections having no primary antibody applied were used to determine DAB staining; these slides did not produce nonspecific DAB staining at 1 minute. Thus, 1 minute was used as the DAB incubation time for slides incubated with CD73 antibody. Stained slides were scanned using an Aperio LVI Real-time Digital Pathology System (Leica Biosystems; Supplementary Fig. S1). IHC for CD3 and PD-L1 was previously performed for the same tumors (14).

### Development of classifications for CD73 staining patterns

Of the 119 tumors that successfully stained for CD73, 15 were randomly selected to develop classifiers for CD73 staining patterns and intensity levels. Whole-slide scanned images (Aperio LVI) were uploaded to Aperio ImageScope (version 12.4, RRID: SCR_020993, Leica Biosystems; Supplementary Fig. S1A and S1B). Classifications were developed for cancer and stromal cell staining patterns, and intensity of CD73 staining was marked as either 1+, denoting positive, low level of CD73 expression or 2+, indicating a high level of CD73 staining (Supplementary Figs. S1C, S2, and S3). Using the developed classifiers, CD73 staining was manually assessed and annotated across the remaining 104 tumors. For all tumors, CD73 staining patterns were annotated across ≥95% of the area of the tumor sections (Supplementary Fig. S1B). Quantification of CD73 staining is reported as percent area of classifier to total area of all classifiers for cancer cells or for stromal cells. The following equation was used for calculating percent of cancer cell staining and for percent of stromal cell staining, respectively. Aperio ImageScope calculates area as μm^2^.

$$\%\;A\text{rea}\;\text{of}\;c\text{lassifier}\;X\;=\;\frac{S\text{um}\;\text{of}\;\text{areas}\;\text{identified}\;\text{as}\;c\text{lassifier}\;X\;}{S\text{um}\;\text{of}\;\text{areas}\;\text{of}\;\text{all}\;c\text{lassifiers}}$$

### Cancer and stromal cell classifications

Eight epithelial cancer cell (Supplementary Fig. S2) and three stromal cell (Supplementary Fig. S3) staining patterns were identified. CD73 cancer cell staining classifications were based on subcellular localization [membrane (apical and/or basolateral) vs. cytoplasm], membrane intensity (1+ = low, downregulation of CD73 expression vs. 2+ = high, normal CD73 expression), and no staining. Subcellular expression patterns for CD73 varied more frequently in the cancer cells, resulting in a larger number of cancer cell classifications. The eight classifications were combined into three representing normal endometrial CD73 staining patterns (membrane + cytoplasm staining), CD73 downregulation (membrane downregulation + cytoplasm staining), and no CD73 staining (Supplementary Fig. S2). Stromal cell CD73 staining was defined as “high” (2+), “low” (1+), and no staining (Supplementary Figs. S2 and S3).

### Cell lines

HEC-1-A wild-type cells (*NT5E*^*+/+*^) were purchased from ATCC (RRID: CVCL_0293, cat. #HTB-112). HEC-1-A cells with CRISPR/Cas9 deletion of *NT5E* (*NT5E*^*−/−*^), also known as CD73-knockout cells, have been previously described elsewhere (17). Cells were maintained in McCoy’s 5A medium supplemented with 10% FBS and 1x penicillin–streptomycin. Cells were subcultured every 3 days at a 1:3 or 1:4 ratio. All experiments were performed using cells between passages 12 and 30. Cell authentication via short tandem repeat sequencing and *Mycoplasma* testing were performed by the University of Texas MD Anderson Characterized Cell Authentication Core within approximately 1 year of experimental use.

### RNA-seq

Total RNA was isolated using QIAshredder and RNeasy Mini Kits and then subjected to stranded bulk mRNA sequencing. Transcriptomic sequences were gathered at 150 bp paired-end reads at or exceeding 35 million reads per replicate. The following analysis was performed on the obtained sequences: mapping by hisat2 (version 2.05; RRID: SCR_0155330), assembly by Stringtie (version 1.3.3b; RRID: SCR_016323), quantification by featureCount (version 1.5.0-p3; RRID: SCR_012919), and DE analysis by DESeq2 (version 1.20.0; RRID: SCR_015687). Gene expression heatmaps were generated by ComplexHeatmap (version 2.8.0; RRID: SCR_025737) using standardized log_2_-transformed Fragments Per Kilobase of transcript per Million mapped reads value.

### Single-cell RNA-seq analysis

Canonical correlation analysis was used to integrate data from different patients. For quality control, only cells with more than 200 and less than 7,500 genes expressed and less than 10% mitochondrial counts were retained. In total, 7,114 cells were used for downstream analyses after the quality control. For finding clusters using Seurat, 20 dimensions of reduction and 0.5 resolution were used. A Uniform Manifold Approximation and Projection (UMAP) plot was also generated using the 20 dimensions of reduction by Seurat (RRID: SCR_016341). The R package cellassign (version 0.99; RRID: SCR_025986) was used to perform cell type assignment. Marker genes used for cell type assignment can be found in Cochrane and colleagues (15). The R package clusterProfiler was used for GO enrichment analysis for the marker genes in each cluster.

### β-Catenin^ΔEX3^ expression and cancer-associated fibroblast assays

The XE49 pt Beta-catenin-myc plasmid, referred to as β-catenin^ΔEX3^ (RRID: Addgene_16840, deposited by Randall Moon), was transfected into HEC-1-A cells at a concentration of 2 μg with and without 25 nmol/L *NT5E* siRNA (5′-CGC​AAC​AAU​GGC​ACA​AUU​ATT-3′) or nontargeting siRNA (Sigma, #SIC001) using Lipofectamine 2000 per the manufacturer’s instructions (Thermo Fisher Scientific). Conditioned media were collected for ELISA assays. Primary human cancer-associated fibroblasts (CAF) from a low-grade EEC (specimen ID: PCD-10-1510 and donor ID: 104655) were purchased from BioIVT, cultured for two passages, and then treated with human recombinant dickkopf-related protein 1 (DKK1; R&D Systems, RRID: SCR_025547, cat. #5439-DK).

### CD73 staining in exon 3 *CTNNB1*-mutant endometrial tumors

Study approval was obtained by the University of Texas MD Anderson Cancer Center’s institutional review board (LAB01-718). Formalin-fixed, paraffin-embedded tissue sections of 11 exon 3 *CTNNB1*-mutant endometrial tumors were obtained from a previously described cohort (19). CD73 IHC was performed as described here (“Materials and Methods” section) and elsewhere (17, 18).

### ELISA, qRT-PCR, and immunoblotting

DKK1 protein levels were measured by ELISA (R&D Systems, RRID: AB_2924151, cat. #DKK100B). RNA was isolated as previously described using TRIzol Reagent (Invitrogen) and chloroform (18). Then 30 ng/μL of DNase-treated RNA was reverse transcribed using a High-Capacity cDNA Reverse Transcription Kit (Applied Biosystems, RRID: SCR_024641, cat. #4374967) and PCR performed using the iTaq Universal Probes One-Step Kit (Bio-Rad, RRID: SCR_024653, cat. #1725140) and a Bio-Rad CFX96 Touch Real-Time PCR Detection System. The following predesigned Taqman primers and probes were purchased from Applied Biosystems: *DKK1* (Hs00931310), *NT5E* (Hs00159686), *CXCL1* (Hs00236937), *CXCL8* (Hs00174103), and *IL6* (Hs00174131). Gene expression was normalized to *HPRT1* (Hs02800695) or 18S (Hs099999901). Immunoblotting for CD73 (Cell Signaling Technology, RRID: AB_2716625, cat. #13160) and myc-tagged β-catenin^ΔEX3^ (Cell Signaling Technology, RRID: AB_490778, cat. #2278) was performed as previously described (17, 18).

### Statistical analysis

*P* values were calculated using two-sided unpaired tests, one-way ANOVA with a Tukey multiple comparisons test, Wilcoxon rank-sum test, or as otherwise indicated. For analyses involving human tissue data, two-sided Fisher exact test, Baptista–Pike odds ratios, and Spearman rank correlation coefficients with corresponding significance tests were used. Correlation matrices and statistical significance were visualized using the R package corrplot (RRID: SCR_024683). Survival analyses were performed using Kaplan–Meier methods. GraphPad Prism (RRID: SCR_002798), SRplot (RRID: SCR_025904), and STRING (version 12.0; RRID: SCR_005223) were used for statistical analysis, data visualization, and graphing.

## Results

### CD73 cancer and stromal cell expression are inversely correlated in low-grade endometrial carcinoma

CD73 is expressed by different cell types in tumors, and cell type–specific expression has been associated with patient outcomes (5, 20, 21). Accordingly, we first developed a comprehensive IHC pipeline to assess CD73 expression in cancer and stromal cells in 119 G2 EEC^MSS^ (Supplementary Figs. S1A–S1C, S2A–S2G, and S3A–S3C), annotating expression intensity and localization across ≥95% of whole-slide tissue sections (Supplementary Fig. S1A and S1B).

**Figure 1.**
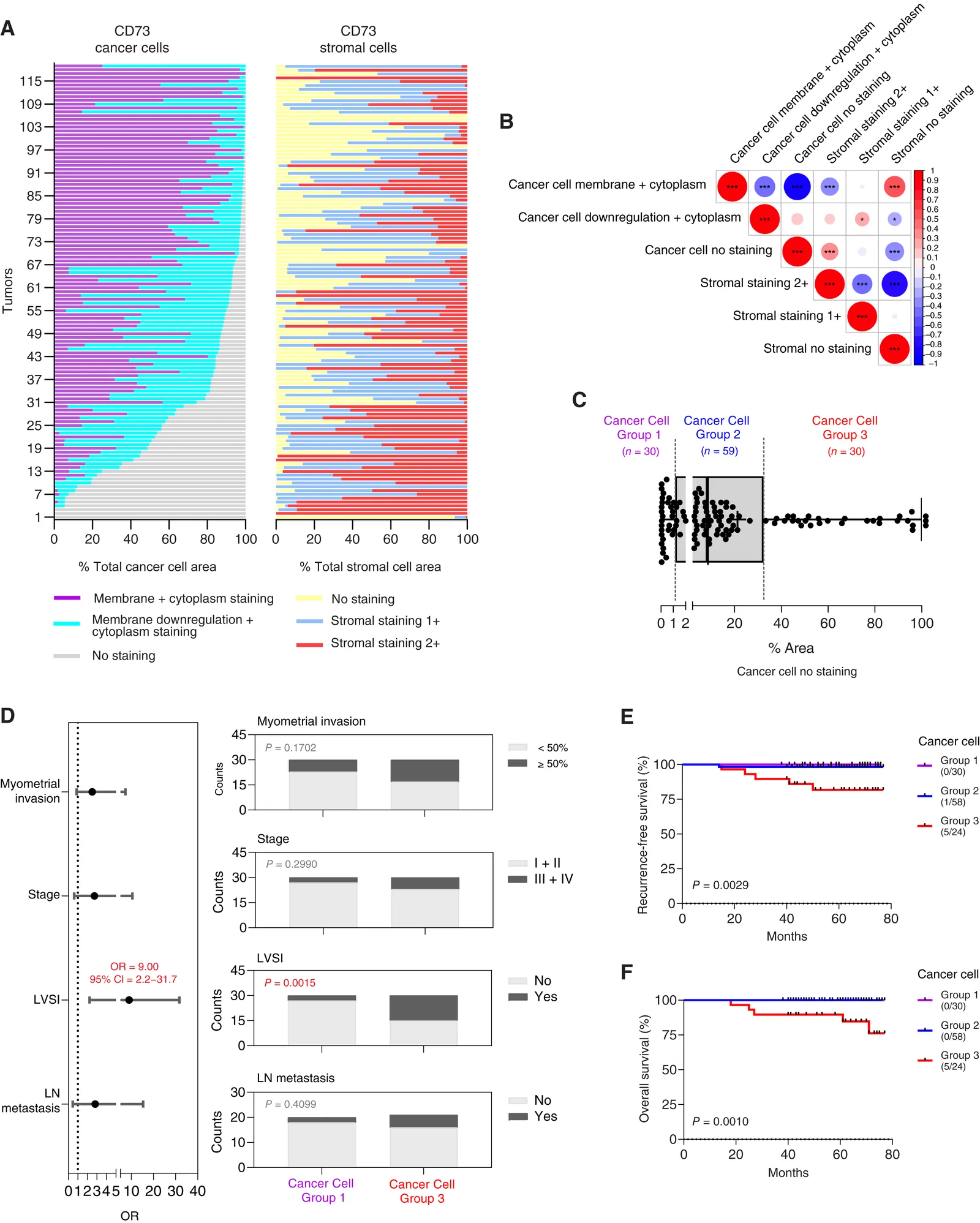
Loss of cancer cell CD73 is associated with aggressive clinical features and poor outcomes. **A,** Expression mapping of CD73 IHC staining categories in cancer and stromal cells across 119 individual G2 EEC^MSS^. Tumors are ordered by decreasing percentage of cancer cell area lacking CD73 staining. Cancer cell staining categories include membrane + cytoplasm staining, membrane downregulation with cytoplasmic staining, and no staining. Stromal CD73 staining is categorized as no staining, stromal staining 1+, or stromal staining 2+. **B,** Spearman correlations comparing CD73 staining patterns in cancer and stromal compartments. Circle color indicates Spearman rho, circle size reflects correlation strength, and asterisks indicate significance (*, *P* < 0.05; ***, *P* < 0.001). **C,** Tumors were stratified into three groups based on quartiles of cancer cell CD73-negative (no staining) area: Group 1, low/no CD73 loss (≤1.15%; *n* = 30); Group 2, intermediate CD73 loss (1.19%–25.68%; *n* = 59); and Group 3, high CD73 loss (32.38%–100%; *n* = 30). The plot displays the distribution of tumors according to the percentage of cancer cell area lacking CD73 staining. Each point represents an individual tumor; the *x*-axis indicates the percentage of cancer cell area with no CD73 staining, and the *y*-axis represents individual tumors ranked by CD73-negative (no staining) area. **D,** Association of CD73 loss with clinicopathologic features comparing Group 1 and Group 3. Features include myometrial invasion, stage, LVSI, and lymph node (LN) metastasis. Bar plots show number of tumors (tumor counts). The Fisher exact test was used for group comparisons, and ORs were calculated using the Baptista–Pike method. CI, confidence interval. **E** and **F,** Recurrence-free and overall survival comparisons in Group 1 vs. Group 3 by the log-rank (Mantel–Cox) test; *P* values shown. **D–F,** Information for clinical features, recurrence-free survival, or overall survival was not available for all tumors.

CD73 expression varied among cancer and stromal cells (Fig. 1A; Supplementary Fig. S4A). Half of the tumors showed either a high percentage of cancer cells with membrane + cytoplasm CD73 staining, which is similar to normal endometrial epithelial cell staining (3) or a high percentage with CD73 loss (no staining; Fig. 1A). Endometrial carcinomas typically grow microscopically as interconnected malignant glands with few intervening stromal cells. We observed that stromal cells accounted for ∼25% of the tumor area (Supplementary Fig. S4A and S4B). Correlation analyses revealed an inverse relationship between cancer and stromal cell CD73 expression (Fig. 1B). Tumors with high cancer cell CD73 were more likely to have low to no stromal CD73 (stromal staining 1+ and no staining), whereas tumors with cancer cell CD73 loss showed increased stromal CD73. The role of stromal cell CD73 in endometrial carcinomas is currently unknown.

### Loss of cancer cell CD73 is associated with increased LVSI and worse patient outcomes

With cancer and stromal cells having distinct CD73 expression patterns, we first assessed the clinical relevance of CD73 cancer cell expression. Tumors were supervised by percent “no staining” for CD73 in the cancer cells and divided into groups based on quartiles (Fig. 1A–C). Cancer Cell Group 3 represents tumors with the greatest loss of cancer cell CD73 expression, and Cancer Cell Group 1 represents tumors with little to no loss (≤1.15%; Fig. 1A–C). Loss of cancer cell CD73 expression in G2 EEC^MSS^ (Cancer Cell Group 3) was associated with lymphovascular space invasion (LVSI; Fig. 1D; Supplementary Fig. S5) and worse survival outcomes (Fig. 1E and F) compared with tumors having little to no loss of cancer cell CD73 expression (Cancer Cell Group 1). These data are similar to CD73 downregulation in high-grade endometrial carcinoma (3) and suggest that loss of cancer cell CD73 is essential for tumor progression in low-grade endometrial carcinoma. LVSI is an independent risk factor for recurrence and worse outcomes in low-risk, early-stage endometrial carcinomas (22) and has been recently linked to immune and inflammatory changes within the tumor microenvironment (23).

### Stromal cell CD73 expression does not associate with clinical features or patient outcomes

We next determined whether strong stromal cell CD73 expression was associated with clinical outcomes. Tumors were supervised by percent stromal staining 2+ and divided into three groups based on quartiles (Supplementary Fig. S6A and S6B). Tumors with the highest percentage of stromal cells (≥55.16%) with strong CD73 staining (Stromal Cell Group 3) did not associate with poor clinical features or patient outcomes compared with tumors with a low percentage (≤5.46%; Stromal Cell Group 1) of stromal staining 2+ (Supplementary Fig. S6C–S6E). Although CD73 cancer cell loss correlates with a higher likelihood of strong stromal CD73 expression (Fig. 1B), these data show that increased stromal expression is not driving poor clinical features or patient outcomes. Rather, loss of cancer cell CD73 is the major contributor to these clinical endpoints.

### Loss of CD73 is associated with the upregulation of pro-inflammatory gene expression

**Figure 2.**
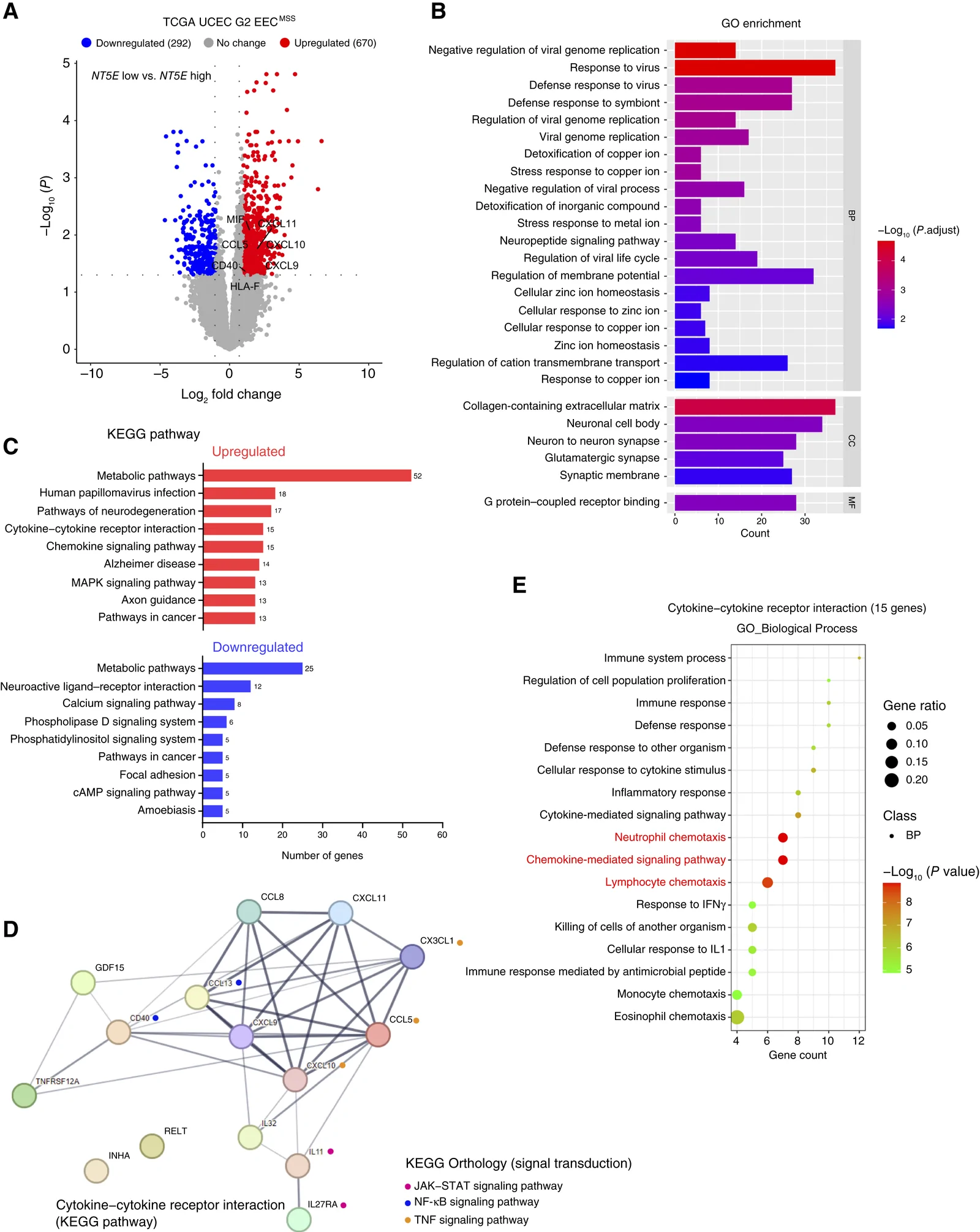
Loss of CD73 is associated with increased pro-inflammatory cytokine/chemokine expression. **A,** Volcano plot showing DEGs in 50 G2 EEC^MSS^ from TCGA-UCEC stratified by low vs. high *NT5E* expression. *NT5E*-low and -high were defined by the lower and upper quartiles, respectively. Red and blue dots indicate genes upregulated and downregulated in *NT5E*-low cases. **B,** GO analysis of DEGs in *NT5E*-low vs. *NT5E*-high tumors. BP, CC, and MF indicate GO Biological Process, Cellular Component, and Molecular Function categories, respectively. **C,** KEGG pathway enrichment analysis of genes upregulated or downregulated in *NT5E*-low cases. Upregulated pathways include cytokine–cytokine receptor interaction and chemokine signaling. **D,** STRING protein–protein interaction network of genes within the cytokine–cytokine receptor interaction pathway identified in **C**. Annotations, indicated by colored circles, are associated immune signaling pathways. **E,** GO Biological Process (BP) enrichment analysis of the 15 cytokine–cytokine receptor interaction genes identified in **C**, showing particular enrichment of neutrophil and lymphocyte chemotaxis and chemokine-mediated signaling.

As an unbiased approach for determining whether CD73 has a role in the immunobiology of low-grade endometrial carcinomas, we assessed the transcriptional landscape of *NT5E*-low versus *NT5E*-high G2 EEC^MSS^, using the TCGA UCEC dataset (11). *NT5E* is the gene that encodes CD73. Figure 2A shows the statistical significance versus magnitude of change of DEGs in *NT5E*-low (lower quartile) compared with *NT5E*-high (upper quartile) G2 EEC^MSS^. GO enrichment revealed that many genes were related to viral responses, particularly triggering defensive responses (Fig. 2B). Further interrogation using Kyoto Encyclopedia of Genes and Genomes (KEGG) pathways revealed that inflammatory factors were among the top genes upregulated in *NT5E*-low G2 EEC^MSS^ (Fig. 2C). Of the 15 genes of the “cytokine–cytokine receptor interaction group” that were upregulated in *NT5E*-low tumors, several (including *CCL5*, *CCL13*, *CXCL9*, *CXCL10*, *CXCL11*, *CD40*, and *IL32*) are known pro-inflammatory factors and/or targets of pro-inflammatory signaling pathways, including JAK–STAT, NF-κB, and TNF [Fig. 2D, KEGG Orthology (signal transduction) and Supplementary Table S2]. Thus, *NT5E*-low tumors exhibit a pro-inflammatory microenvironment. Notably, in *NT5E*-low tumors, genes related to signaling pathways downstream of the adenosine receptors, such as cyclic adenosine monophosphate, phosphatidylinositol, and calcium signaling, were among the most downregulated (Fig. 2C), consistent with loss of CD73/extracellular adenosine signaling.

In response to viral infections, pro-inflammatory cytokines and chemokines are essential to activating innate immune cell responses as defense mechanisms to eliminate infected cells. These include increasing the expression of costimulatory molecules and chemoattractants for activating innate immune cells and subsequent development of adaptive immunity (24, 25). Accordingly, we assessed the biological process of the 15 genes of the “cytokine–cytokine receptor interaction group” (Fig. 2C) to better understand their immune roles. Nearly half of these genes were known to promote neutrophil (seven genes) and lymphocyte (six genes) chemotaxis (Fig. 2E). A smaller number (four of 15 genes) promoted monocyte and eosinophil chemotaxis (Fig. 2E), and several genes were related to responses of IFNγ (five genes), a classic pro-inflammatory mediator. IFNγ is known to induce *CXCL9*, *CXCL10*, and *CXCL11*, which promote effector T-cell and NK-cell trafficking (26) and CD8^+^ T-cell recruitment into tumors (27). Together, these data suggest that CD73 loss promotes a pro-inflammatory microenvironment with the potential to promote antitumor immune responses.

### Pro-inflammatory gene expression correlates with increased immunosuppressive gene expression in *NT5E*-low tumors

**Figure 3.**
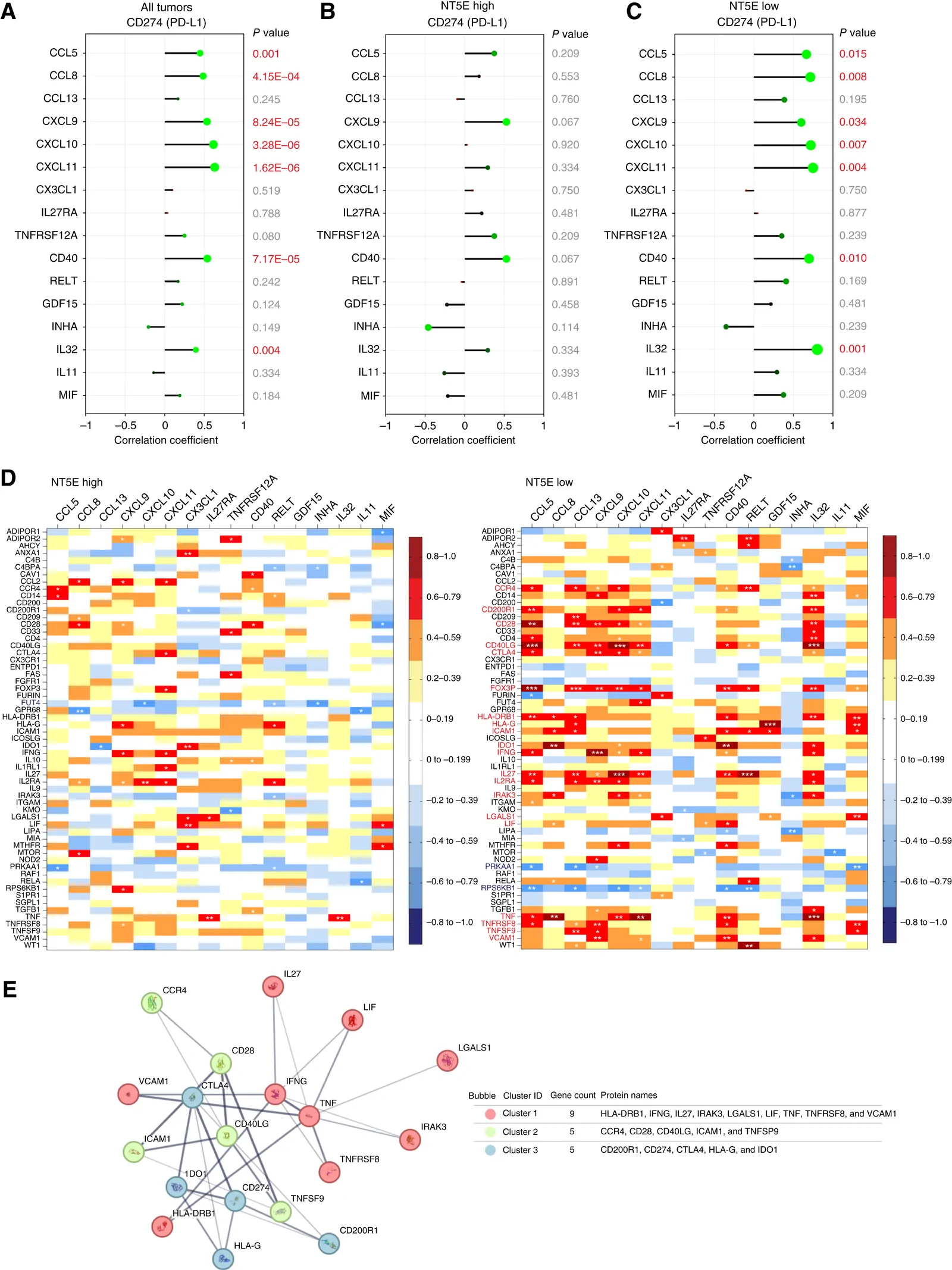
Pro-inflammatory cytokine/chemokine expression correlates with increased PD-L1 and immunosuppression gene expression in *NT5E*-low tumors. **A–C,** Spearman correlations of *CD274* (PD-L1) vs. the 15 cytokine–cytokine receptor interaction genes identified in Fig. 2C. Correlations are shown in G2 EEC^MSS^ for (**A**) all tumors, (**B**) *NT5E*-high tumors, and (**C**) *NT5E*-low tumors. *NT5E*-low and -high tumors were those defined by the lower and upper quartiles in Fig. 2A. **D,** Spearman correlations of immunosuppression genes vs. the 15 cytokine–cytokine receptor interaction genes identified in Fig. 2C in *NT5E*-high and *NT5E*-low cases. The list of immunosuppressive genes was from Harmonizome 3.0, Immunosuppression. **E,** STRING protein–protein interaction network of immunosuppressive genes with ≥3 significant correlations, corresponding to genes highlighted in red or blue in **D**, clustered by K-means. *, *P* < 0.05; **, *P* < 0.009; ***, *P* < 0.0009.

We next examined whether *NT5E*-low tumors counter tumor-constraining inflammation by upregulating immunosuppressive genes. We first focused on *CD274* (encoding PD-L1), a key immunosuppressive mediator. We assessed correlations between *CD274* and the 15 upregulated “cytokine–cytokine receptor interaction” genes (Fig. 2C; Supplementary Table S2). Among these, *CCL5*, *CCL8*, *CXCL9*, *CXCL10*, *CXCL11*, *CD40*, and *IL32* positively correlated with *CD274* expression in all G2 EEC^MSS^ (Fig. 3A).

To test whether these correlations were related to CD73 loss, we compared *NT5E*-high (upper quartile; Fig. 3B) and *NT5E*-low (lower quartile; Fig. 3C) tumors. The same pro-inflammatory genes correlated with *CD274* in *NT5E*-low tumors (Fig. 3C) but not in *NT5E*-high tumors (Fig. 3B). Thus, although CD73 loss promotes a pro-inflammatory microenvironment, *NT5E*-low tumors counter the inflammation by upregulating immunosuppressive genes. Supporting this, several immunosuppressive genes (*CCR4*, *CD200R1*, *CD28*, *CTLA4*, *FOXP3*, and *HLA-G*; Fig. 3D; Supplementary Table S3; 19 genes total) were positively correlated with the same pro-inflammatory genes in *NT5E*-low tumors. These correlations were largely absent in *NT5E*-high tumors (Fig. 3D). K-means clustering of the 19 genes identified key immunosuppressive mechanisms: cluster 1, T-regulatory cell regulation; cluster 2, cytokines (IL4 and IL10) limiting inflammation; and cluster 3, induction of immune tolerance (Fig. 3E).

### Loss of cancer cell CD73 defines a distinct inflamed tumor phenotype characterized by active T-cell infiltration coupled with adaptive immune suppression

**Figure 4.**
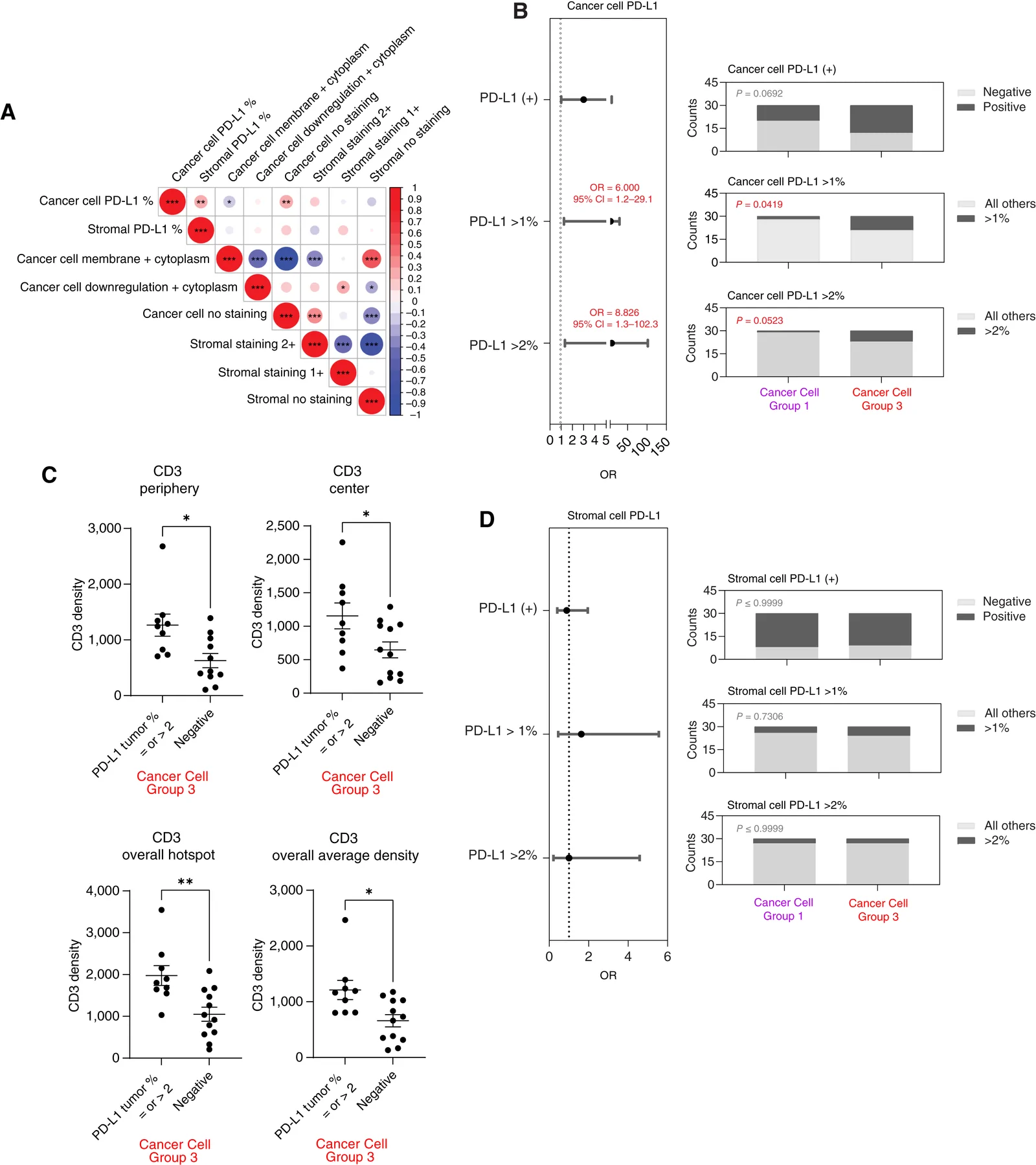
Cancer Cell Group 3 exhibits CD73 loss–associated PD-L1 upregulation and increased T-cell infiltration. **A,** Spearman correlations of cancer and stromal CD73 staining with percent PD-L1 in 119 G2 EEC^MSS^. Circle color indicates Spearman rho, circle size reflects correlation strength, and asterisks indicate significance (*, *P* < 0.05; **, *P* < 0.01; ***, *P* < 0.001). **B** and **D,** Comparison of cancer and stromal PD-L1 expression in Cancer Cell Groups 1 and 3, as defined in Fig. 1C. PD-L1 (+) was defined as any staining, with additional cutoffs shown for >1% and >2% staining. The Fisher exact test was used and ORs estimated by the Baptista–Pike method. **C,** CD3 staining in Group 3 tumors, indicating periphery, center, hotspot, and overall average density, as described by Crumley and colleagues (14). Welch *t* test: periphery *, *P* = 0.01; center *, *P* = 0.02; hotspot **, *P* = 0.003; and overall *, *P* = 0.01. CI, confidence interval.

To independently validate that tumors with CD73 loss were more likely to have increased PD-L1, we examined PD-L1 protein expression in our G2 EEC^MSS^ IHC cohort (Fig. 1). Consistent with *CD274* expression in the TCGA-UCEC data for G2 EEC^MSS^ in Fig. 3C, we found that tumors with cancer cell CD73 loss (no staining) showed a higher percentage of PD-L1–positive cancer cells, whereas tumors with high membrane + cytoplasm CD73 staining exhibited lower PD-L1 expression (Fig. 4A). These data were secondarily confirmed using the Cancer Cell Groups described in Fig. 1C. Tumors with a high percent of cancer cell CD73 loss (Cancer Cell Group 3) were more likely to have an increased percentage of cancer cells expressing PD-L1 (PD-L1 >1% and PD-L1 >2%; Fig. 4B). Together, these data support a link between cancer cell CD73 loss and tumor-intrinsic immune checkpoint upregulation in low-grade EECs.

To assess whether this phenotype reflects an immune-inflamed microenvironment, we examined CD3^+^ T-cell infiltration. High cancer cell PD-L1 expression was associated with increased CD3^+^ immune infiltration, particularly in Cancer Cell Group 3 tumors (Fig. 4C; Supplementary Fig. S7A and S7B), across multiple assessments, including the tumor periphery, center, hotspot regions, and overall average density in G2 EEC^MSS^. By contrast, CD8^+^ cell density did not differ between cancer cell CD73 groups (Supplementary Fig. S8A–S8C). Thus, cancer cell CD73 loss is associated with increased total T-cell infiltration without a corresponding increase in cytotoxic CD8^+^ cells. In many cancers, the combination of T-cell infiltration and PD-L1 expression is indicative of adaptive immune resistance (28, 29). Accordingly, the absence of increased CD8^+^ T-cell density suggests that the expanded CD3^+^ infiltrate may include noncytotoxic or immune-regulatory T-cell populations. Together, these data support that CD73 loss defines a distinct immune-engaged tumor phenotype characterized by increased T-cell infiltration, PD-L1 expression, and features of adaptive immune suppression.

Given that CD73 expression increases in stromal cells of tumors with cancer cell CD73 loss (Fig. 1A and B), we examined PD-L1 expression in stromal cells. Strong stromal CD73 (stromal staining 2+) did not correlate with PD-L1 in cancer or stromal cells (Fig. 4A), and stromal cell PD-L1 expression was independent of cancer cell CD73 status (Group 1 vs. Group 3; Fig. 4D). These data indicate that stromal cell CD73 does not drive cancer cell PD-L1 expression and that stromal PD-L1 is independent of CD73 status.

### *NT5E* deletion in low-grade endometrial cancer cells increases inflammatory and immunosuppressive gene expression

**Figure 5.**
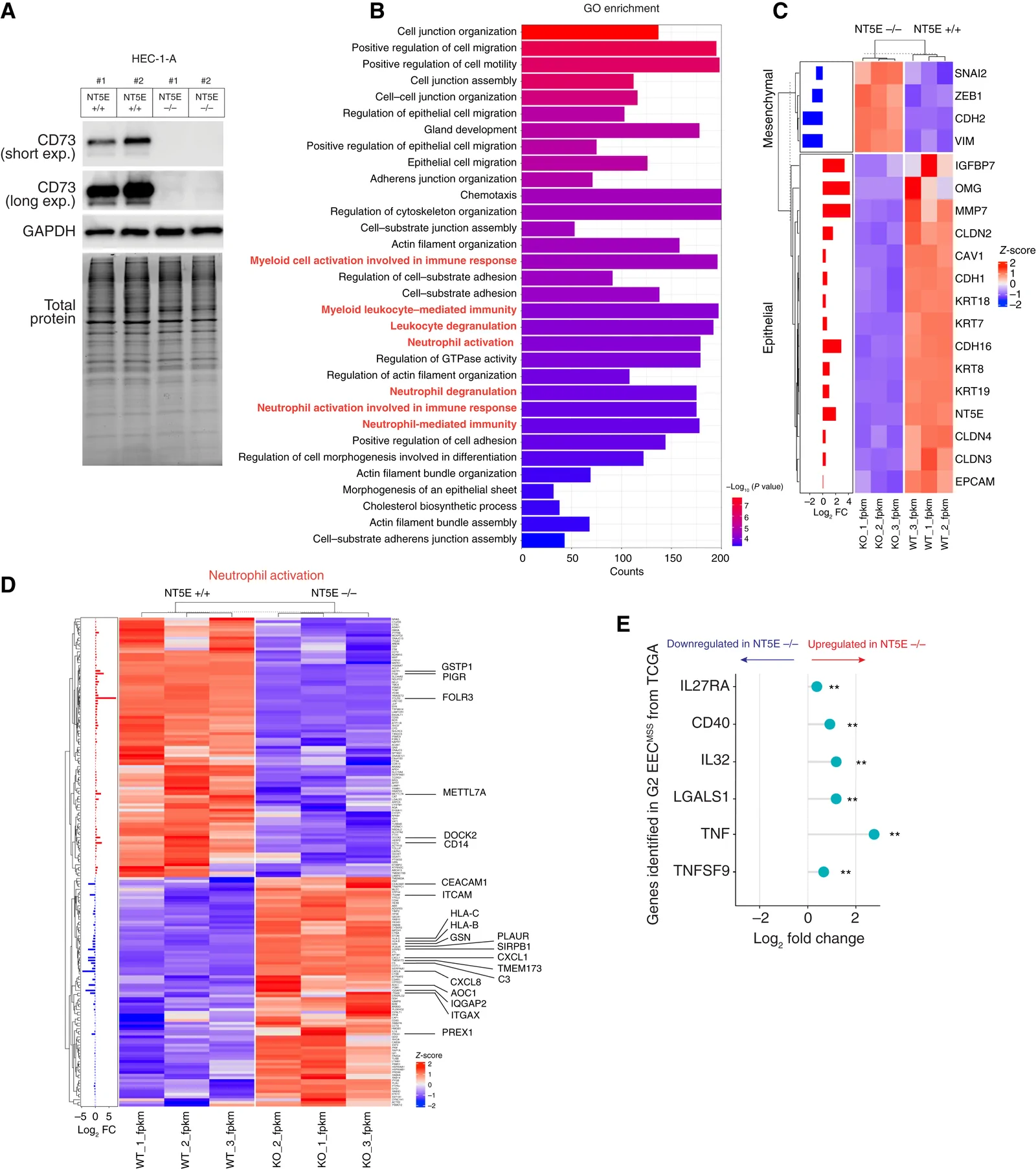
*NT5E* deletion reduces epithelial integrity and increases pro-inflammatory and immunosuppression gene expression. **A,** Immunoblot validation of *NT5E* deletion by CRISPR/Cas9 in HEC-1-A cells, shown for replicate samples #1 and #2. GAPDH and total protein staining are shown as loading controls. **B,** GO enrichment analysis of DEGs in *NT5E*^*+/+*^ vs. *NT5E*^*−/−*^ cells, with innate immune pathways highlighted in red. **C,** Epithelial and mesenchymal gene expression in *NT5E*^*−/−*^ vs. *NT5E*^*+/+*^ cells. Red indicates increased expression and blue indicates decreased expression by *Z*-score. **D,** Heatmap of neutrophil activation genes identified in **B**, with genes showing ≥2 log_2_ fold change (FC) highlighted. **E,** Representative genes upregulated in HEC-1-A *NT5E*^*−/−*^ vs. *NT5E*^*+/+*^ that overlap with upregulated cytokine–cytokine receptor interaction and immunosuppressive genes identified in *NT5E*-low G2 EEC^MSS^ in Figs. 2C and 3D. **, *P* < 0.005.

To functionally validate that loss of cancer cell CD73 results in the upregulation of inflammatory genes, we profiled the transcriptome of low-passage (P12) isogenic *NT5E*^*−/−*^ and *NT5E*^+/+^ HEC-1-A cells (Fig. 5A) using RNA-seq. GO analyses revealed that genes essential to epithelial integrity were among the most DEGs (Fig. 5B and C), corroborating previous studies that CD73 is essential in maintaining endometrial epithelial integrity (3, 18).

Similar to *NT5E*-low G2 EEC^MSS^ (Fig. 2), genes involved in innate immune activation—including neutrophil and myeloid cell activation—were prominently altered in *NT5E*^*−/−*^ HEC-1-A cells [Fig. 5B (red highlighted text) and D]. Upregulated neutrophil activation genes included cancer cell–expressed or –secreted factors that recruit immune cells and promote antitumor responses (*HLA-B*, *HLA-C*, *GSN*, *CXCL1*, and *CXCL8*; Fig. 5D; Supplementary Table S4). We next examined whether cytokine–cytokine receptor interaction genes identified in *NT5E*-low tumors in Fig. 2C were similarly altered in *NT5E*^*−/−*^ HEC-1-A cells.

Increased expression of *IL2RA*, *CD40*, and *IL32* was observed in *NT5E*^*−/−*^ compared with *NT5E*^*+/+*^ cells (Fig. 5E). Additionally, genes that mediate both pro-inflammatory and immunosuppressive responses, such as *LGALS1*, *TNF*, and *TNFSF9*, which were previously observed in *NT5E*-low tumors in Fig. 3D, were found upregulated in *NT5E*^*−/−*^ compared with *NT5E*^*+/+*^ HEC-1-A cells (Fig. 5E; Supplementary Table S3). Together, these data functionally show that cancer cell CD73 loss promotes the expression of pro-inflammatory and immunosuppressive genes. *CD274* expression in *NT5E*^*−/−*^ HEC-1-A cells showed a *P* value of 0.02 and an adjusted *P* value of 0.09, consistent with a trend toward PD-L1 upregulation with CD73 loss.

### Single-cell analysis reveals rare co-expression of CD73 and PD-L1 in endometrial carcinoma cells

**Figure 6.**
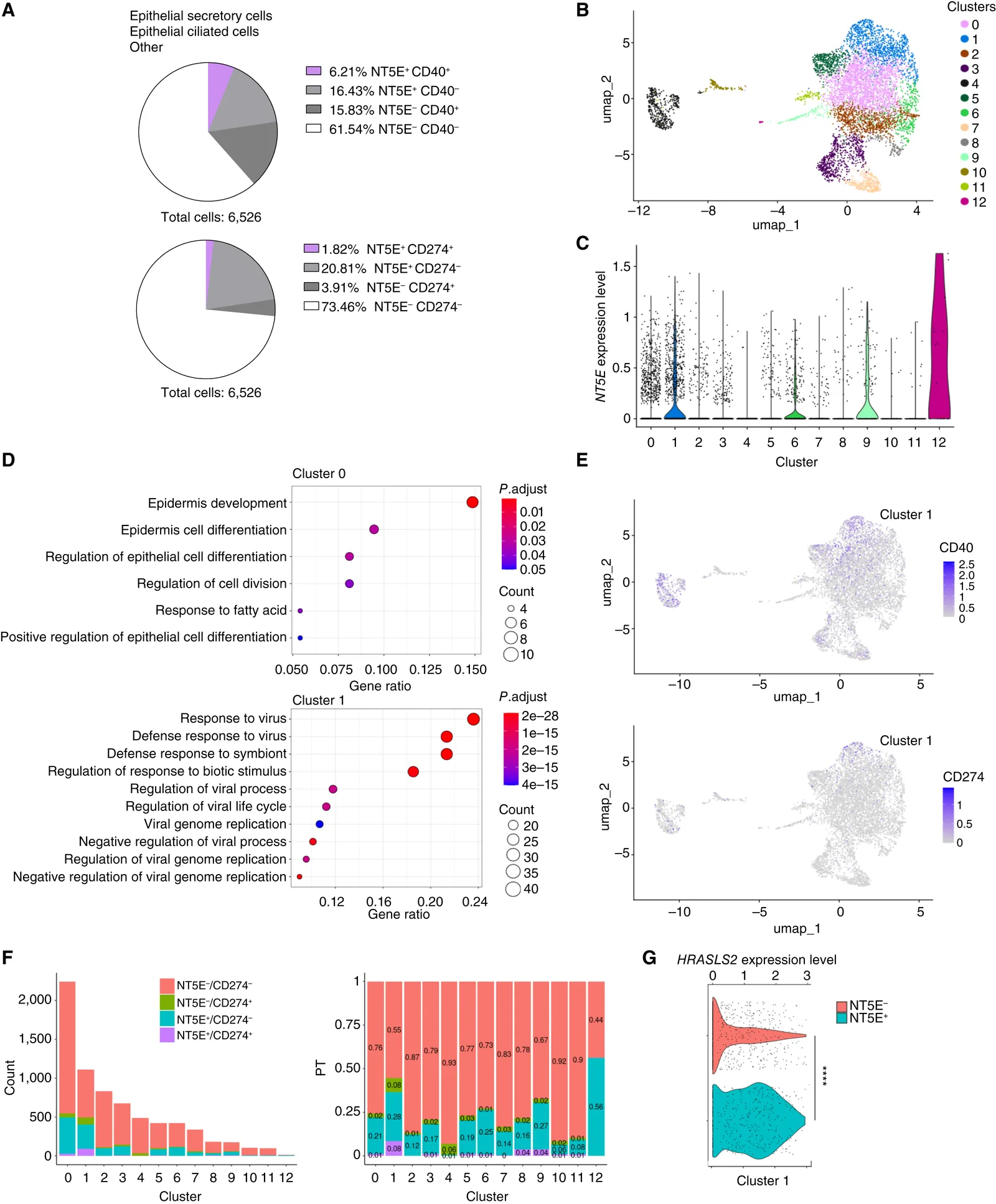
CD73–PD-L1 co-expression is rare and is inversely associated in immune-responsive cells. **A,** Single-cell expression of *CD40* and *CD274* in nonimmune cells in six endometrial tumors. Pie charts show the proportion of cells with positive or negative expression of each marker. **B,** Integrated Uniform Manifold Approximation and Projection of 13 transcriptionally distinct clusters using Seurat. **C,***NT5E* expression across clusters. **D,** GO enrichment analysis of individual clusters. Enrichment plots shown are for clusters 0 and 1, in which *NT5E* is frequently and highly expressed. Cluster 0 shows pathways associated with epithelial differentiation, and cluster 1 shows pathways related to immune response. **E,** Uniform Manifold Approximation and Projection plots showing *CD40* and *CD274* (PD-L1) expression across clusters. **F,** Stacked bars are showing the distribution of *NT5E-* and *CD274*-positive and -negative cells across clusters. Positive (+) expression for all genes was defined as values above 0; negative (−) expression was defined as 0. Stacked bars in the graph to the right show the proportion of *NT5E/CD274* expression groups within each cluster. **G,***NT5E+* cells are associated with increased *HRASLS2* expression in cluster 1 (Wilcoxon, ****P < 0.0001). UMAP, Uniform Manifold Approximation and Projection.

To assess whether the inflammatory signature in *NT5E*^*−/−*^ HEC-1-A cells and *NT5E*-low tumors occurs at the single-cell level, we analyzed a publicly available single-cell RNA-seq dataset of high- and low-grade endometrial tumors (15). *NT5E* expression was largely restricted to cancer cells, with a few normal epithelial cells (secretory and ciliated) and “other” cells present, which likely represent fibroblasts or epithelial marker–negative cancer cells (Supplementary Fig. S9A–S9C). By contrast, *NT5E* expression was minimal or undetectable across immune cell populations, suggesting that immune cells are unlikely to be a major source of CD73 in this tumor microenvironment (Supplementary Fig. S9C). Consistent with CD73 loss promoting inflammation, *NT5E*-positive nonimmune cells were three times more likely to be *CD40*-negative (16.43%) than *CD40*-positive (6.21%) and ∼11 times more likely to be *CD274*-negative than positive (20.81% vs. 1.82%; Fig. 6A).

### A distinct immune-responsive cluster shows inverse association between CD73 and PD-L1

We next performed cluster analysis to examine the association of *NT5E* expression with different cell populations. Thirteen transcriptionally distinct clusters were identified (integrative data, Fig. 6B; individual tumor data, Supplementary Fig. S10A), with *NT5E* variably expressed across several nonimmune clusters (clusters 0, 1, 6, 9, and 12; Fig. 6C). Clusters reflected distinct biological programs: cluster 0 was enriched for genes regulating epithelial and epidermal differentiation (Fig. 6D), consistent with CD73’s role in maintaining epithelial integrity (3, 18); cluster 9 featured genes for cilium organization and normal epithelial function (Supplementary Fig. S10B); cluster 6 showed no enrichment; and cluster 12 was defined by genes linked to reproductive function (Supplementary Fig. S10B).

Cluster 1 was characterized by interferon-stimulated antiviral response genes *IFIT1*, *IFIT2*, and *IFIT3* (interferon-induced protein with tetratricopeptide repeats; ref. 30). Their known immunomodulatory roles are consistent with an immune-responsive cluster that is potentially balancing pro-inflammatory and immunosuppressive responses. Consistent with this, *CD40* and *CD274* were frequently expressed in cluster 1, with *CD274* predominantly expressed in this cluster (Fig. 6E). *NT5E*-positive cells in cluster 1 were three times more likely to be *CD274*-negative (Fig. 6F) and were more likely to be associated with viral response genes shown to have tumor suppressor functions, such as *HRASLS2* (Fig. 6G; ref. 31), which also marked cluster 1. The proportions of *IFIT1-*, *IFIT2-*, and *IFIT3-*positive and -negative cells differed only modestly by *NT5E* status (Supplementary Fig. S11A–S11C).

### Loss of cancer cell CD73 in *CTNNB1*-mutant endometrial carcinoma shapes a dual inflammatory and immunosuppressive microenvironment

To examine how CD73 loss might shape a dual inflammatory and immunosuppressive environment, we perturbed CD73 in *CTNNB1* (β-catenin)-mutant endometrial carcinoma cells. Exon 3 *CTNNB1* mutations are common in low-grade EEC and identify a subset with increased risk of recurrence and reduced disease-free survival (9, 32), whereas oncogenic β-catenin promotes invasiveness and cancer cell stemness (17). Here, we asked whether CD73 loss increases β-catenin target genes with known immunomodulatory functions. We focused on DKK1, a secreted Wnt antagonist and direct β-catenin target gene (33) that is upregulated in β-catenin–mutant endometrial carcinoma (34). DKK1 has context-dependent inflammatory and immunosuppressive roles. In nonimmune cells, DKK1 can promote the expression of pro-inflammatory cytokines and chemokines, including IL1β, IL6, and CXCL8 (35). DKK1 has also been shown to promote myeloid-mediated immunosuppression in cancer, including expansion and activation of myeloid-derived suppressor cells and immunosuppressive polarization of macrophages, thereby limiting CD8^+^ T-cell and NK-cell antitumor responses (36, 37).

**Figure 7.**
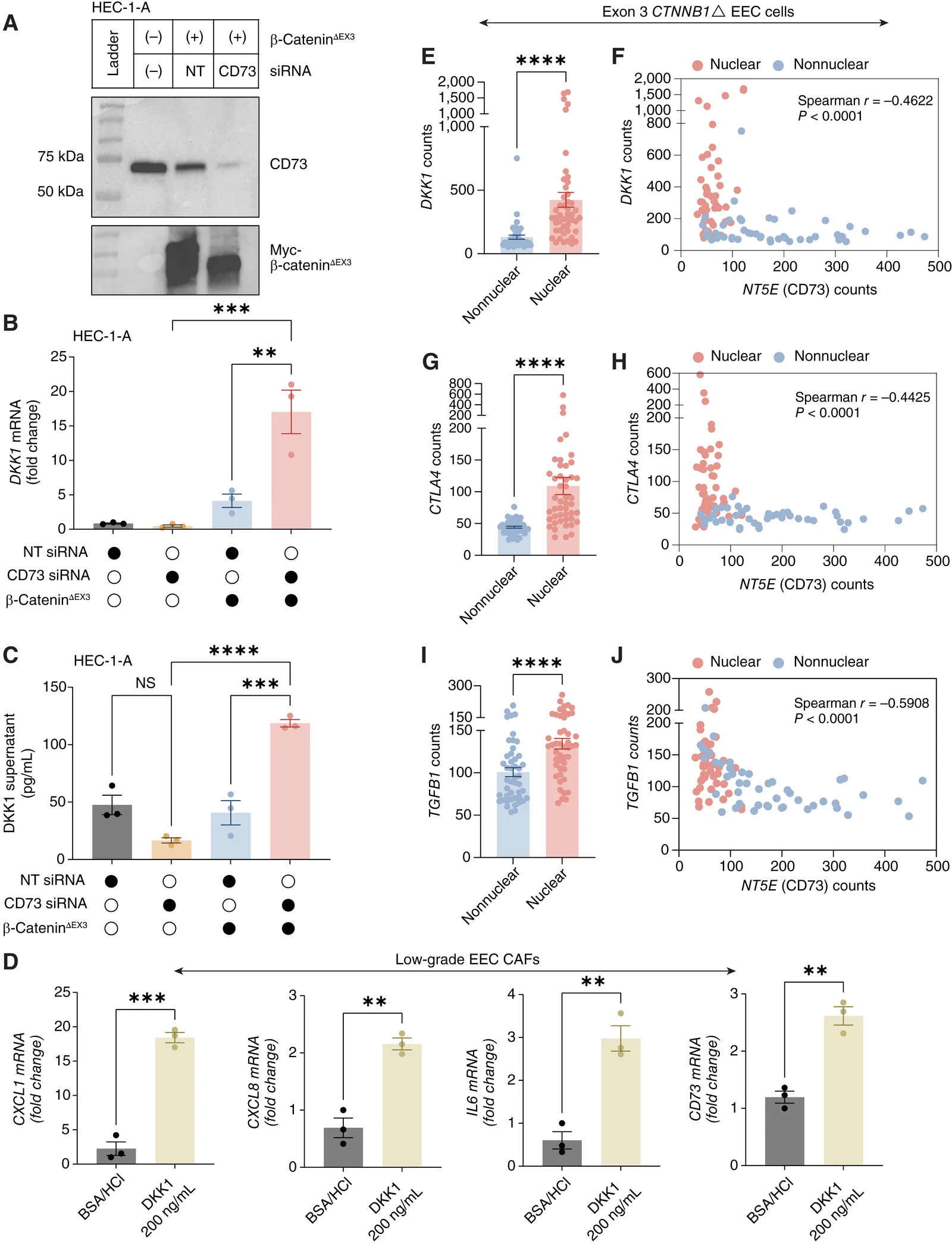
Loss of cancer cell CD73 increases inflammatory mediator DKK1 and correlates with an immune-responsive microenvironment. **A,** Immunoblot validation of exon 3–mutant β-catenin (β-catenin^ΔEX3^) expression and CD73 knockdown in HEC-1-A cells, modeling low-grade EEC. **B** and **C,***DKK1* expression and protein secretion by ELISA in HEC-1A cells ± β-catenin^ΔEX3^ and ±CD73. **D,** Pro-inflammatory cytokine and *NT5E* expression in patient-derived CAFs treated with recombinant DKK1. **E–J,** GeoMx spatial profiling of 16 exon 3 *CTNNB1*-mutant EEC, with 2 to 3 regions of interest analyzed per nuclear and nonnuclear β-catenin regions per tumor, as previously described in (16, 17). Data are shown as mean ± SEM. **B** and **C,** One-way ANOVA with a Tukey test; (**D**) *t* test; and (**E**, **G**, and **I**) Mann–Whitney test. *, *P* < 0.05; **, *P* < 0.005; ***, *P* < 0.0005; ****, *P* < 0.0001. NT, nontargeting siRNA.

Silencing CD73 in HEC-1-A cells expressing mutant β-catenin induced a three- to fourfold increase in DKK1 expression and secretion, whereas mutant β-catenin alone had no effect (Fig. 7A–C). Consistent with DKK1 being a secreted protein with cytokine-inducing activity, recombinant DKK1 increased *IL6*, *CXCL1*, and *CXCL8* expression in primary, low-grade EEC CAFs (Fig. 7D). DKK1 treatment also increased *NT5E* expression in CAFs (Fig. 7D), which is consistent with elevated stromal cell CD73 in exon 3 *CTNNB1*-mutant tumors with cancer cell CD73 loss (Supplementary Fig. S12) and that observed in G2 EEC^MSS^ (Fig. 1A and B).

As validation, we analyzed a publicly available GeoMx spatial profiling dataset of nuclear and nonnuclear regions of β-catenin protein expression in cancer cells from 16 exon 3 *CTNNB1*-mutant EECs (16). DKK1 expression was higher in nuclear β-catenin regions (Fig. 7E), and correlations with *NT5E* showed a robust L-shaped, threshold-dependent relationship (Fig. 7F), indicating that CD73 loss permits strong DKK1 transcription. Given that DKK1 can also promote immunosuppression, we examined spatial correlations between DKK1 and immunosuppressive gene expression. Although *CD274* data were unavailable, *CTLA4*—which we previously found correlated with pro-inflammatory genes in *NT5E*-low G2 EEC^MSS^ (Fig. 3D)—was found elevated in nuclear regions and negatively correlated with *NT5E* (Fig. 7G and H). Similarly, *TGFβ1* and *TGFβ2*, immunosuppressive cytokines (38), were increased in nuclear regions and inversely associated with *NT5E* (Fig. 7I and J; Supplementary Fig. S13A and S13B). Together, these data identify a clinically relevant association between cancer cell CD73 loss, increased DKK1 expression, and a mixed inflammatory and immunosuppressive environment in low-grade EEC.

## Discussion

Tumors evade antitumor immune cells in multiple ways. Using multiple human tumor cohorts and CD73 deletion in endometrial carcinoma cells, we show that loss of cancer cell CD73 increases pro-inflammatory cytokine expression while also marking an immunosuppressive microenvironment in low-grade EEC. Thus, in contrast to other solid tumors in which CD73 overexpression is commonly linked to immune evasion, our findings suggest that CD73 loss may also be as immunologically consequential in endometrial carcinoma.

An unexpected finding of our transcriptomic analyses was the enrichment of pathways annotated as viral infection or antiviral responses in *NT5E*-low human tumors. These annotations do not necessarily indicate that viral infection or CD73 loss induces viral programs. Rather, similar transcriptional responses can be activated by endogenous mechanisms, including interferon signaling, cytosolic nucleic acid sensing, and derepression of endogenous retroelements (39–41). These mechanisms can also induce inflammatory cytokines and chemokines (42, 43), consistent with the enrichment of chemokine signaling and cytokine–cytokine receptor interaction pathways observed in our study. *IFIT* expression did not vary substantially with *NT5E* status in our single-cell analyses, suggesting that CD73 loss does not broadly induce a canonical antiviral response. Together, these findings suggest that CD73 loss activates an endogenous inflammatory program with transcriptional features that partially overlap with antiviral response pathways.

CD73 loss associating with viral defensive/pro-inflammatory response in low-grade EECs aligns with the known consequences of epithelial barrier disruption, especially in intestinal epithelial cells (44). To eliminate pathogen-infected cells in the gut, disruption of the epithelial barrier induces a pro-inflammatory response, analogous in many ways to immune-mediated clearance of abnormal self-cells such as cancer cells. Similar to the gut (45), CD73 is critical to maintaining endometrial epithelial barrier integrity, and we have shown that this homeostatic response remains intact in low-grade EEC (3).

Consistent with the idea that CD73 loss in low-grade EEC triggers an inflammatory response through disruption of epithelial integrity, our data showed that CD73 loss in HEC-1-A cells decreased the expression of epithelial integrity genes while concurrently increasing inflammatory gene expression. Additionally, cytokines and chemokines known to drive pro-inflammatory responses following epithelial barrier disruption in the gut (46–49) were found upregulated with CD73 loss in G2 EEC^MSS^. Therefore, CD73 loss in low-grade EEC likely resembles an inherent, normal endometrial tissue response to the breakdown of the epithelial barrier. Pregnancy represents one physiologic setting in which endometrial epithelial remodeling is coupled with coordinated inflammatory and immune-regulatory responses. Several of the chemokines/cytokines that increased with CD73 loss, including *CXCL9*, *CXCL10*, *CXCL11*, *IL32*, and *CD40*, have been implicated in inflammatory immune signaling at the maternal–fetal interface and in pregnancy-associated inflammatory states (50–53).

During early pregnancy, implantation-associated epithelial remodeling is accompanied by coordinated pro-inflammatory signaling and local immune tolerance programs that support trophoblast invasion while limiting maternal anti-fetal immune responses. Here, endometrial epithelial cells lose cell–cell adhesions and acquire epithelial-to-mesenchymal transition–like features (54), which is accompanied by simultaneous induction of pro-inflammatory cytokines (e.g., IL1β, IL6, and CXCL8) and immunoregulatory mediators (e.g., TGFβ, PD-L1, IDO1, and HLA-G), permitting tissue invasion while maintaining immune tolerance (55–57). In *NT5E*-low G2 EEC^MSS^, multiple factors (e.g., *CD274*, *CTLA4*, *IDO1*, *CCR4*, and *IFNG*), which promote immunosuppressive and immune tolerance responses, were positively correlated with several pro-inflammatory genes (*CCL5*, *CCL13*, *CXCL9*, *CXCL10*, *CXCL11*, *CD40*, and *IL32*). Accordingly, given that CD73-associated homeostatic mechanisms remain intact in low-grade/early-stage EECs (3, 18), it is also possible that CD73 loss activates tissue-inherent immune tolerance programs normally engaged at the maternal–fetal interface. Thus, the immune and transcriptional features associated with CD73 loss in low-grade EEC likely arise from multiple processes, including disruption of CD73-dependent epithelial homeostasis, tissue-intrinsic pregnancy-associated programs, and reduced adenosine-mediated immunosuppression.

In other solid tumors, CD73 expression/overexpression is positively associated with PD-L1 and poor prognosis (58–61). With two independent tumor cohorts of low-grade EEC, we did not observe a positive correlation between PD-L1 and CD73-high expression in cancer cells or stromal cells. Instead, cancer cell CD73 loss correlated with increased PD-L1 expression and poor survival. Low-grade EECs are generally more indolent than high-grade EECs and serous endometrial carcinomas, which are clinically aggressive histotypes. Therefore, the association between cancer cell CD73 loss and poor survival in low-grade tumors highlights its potential contribution to more aggressive low-grade EECs. We have previously shown that CD73 loss in endometrial carcinoma cell lines, including HEC-1-A and Ishikawa cells, models of low-grade endometrial carcinoma, promotes cancer cell migration and invasion *in vitro* (3, 17). We have also demonstrated that CD73 loss in specific subsets of low-grade EEC is associated with disease recurrence (17).

Cancer Cell Group 3—the group with the highest loss of cancer cell CD73—was the only group which T-cell infiltration was associated with tumors showing increased percentage of PD-L1. A limitation of our study is detailed analyses of these T cells. The increase in CD3^+^ T-cell infiltration in the absence of a corresponding increase in CD8^+^ cell density suggests that the expanded T-cell infiltrate is not primarily composed of cytotoxic CD8^+^ T cells. Consistent with this observation, our bulk transcriptomic analyses demonstrated increased expression of *FOXP3*, *CTLA4*, *IL2RA*, and *IDO1* in tumors with cancer cell CD73 loss. Together, these findings raise the possibility that CD73-low tumors are enriched for regulatory and/or functionally exhausted T-cell populations. However, because these analyses were performed on bulk tumor tissue, the cellular source of these transcripts cannot be definitively determined; therefore, single-cell or spatial profiling studies will be required to confirm the identity of the infiltrating T-cell subsets. Nevertheless, our findings show that cancer cell CD73 loss defines a subset of low-grade EEC characterized by PD-L1 expression, heightened immune infiltration, and an immunosuppressive transcriptional program. Although PD-L1–positive cells represented only a small fraction of the tumors in our study, the concurrent increase in CD3^+^ T-cell infiltration, elevated immune regulatory markers, and increased PD-L1 expression with cancer cell CD73 loss suggest activation of adaptive immune-regulatory pathways rather than an isolated immune marker change. Consistent with the clinical relevance of low-level PD-L1 expression, a 1% tumor cell cutoff is used in non–small cell lung cancer (62), whereas combined positive score ≥1 is used in head and neck and cervical cancers (63, 64).

We observed increased stromal CD73 expression accompanying cancer cell CD73 loss. In other tumors, noncancer CD73 promotes immune evasion (21, 65). In our IHC cohort, strong stromal CD73 did not correlate with poor outcomes or PD-L1 expression. Future studies will be necessary to interrogate whether stromal cell CD73 promotes immunosuppression regardless of PD-L1 status. Additionally, given the role of CD73 to promote normal homeostatic programs in low-grade EEC (3, 18), it will also be important to determine whether increased stromal cell CD73 expression represents a tissue-intrinsic pregnancy-associated program. Specifically, increased stromal cell CD73 expression in low-grade EEC with cancer cell CD73 loss and DKK1 treatment may reflect a decidualization-like response. During decidualization, endometrial stromal cells differentiate into decidual cells. These cells gain epithelial-like characteristics as the endometrium undergoes controlled remodeling during early pregnancy and implantation (66). We previously showed that WISH cells, a human amnion-derived cell line, transition from a stromal-like to an epithelial-like state and that CD73 expression increases with epithelialness (3). We also note that DKK1 is increased by progesterone during pregnancy, which helps facilitate the transformation of endometrial stromal cells into decidual cells (67, 68). Our data showed that DKK1 expression and secretion increase with cancer cell CD73 loss. Accordingly, increased stromal CD73 accompanying cancer cell CD73 loss may reflect physiologic endometrial remodeling in response to an inflammatory microenvironment. Because high stromal CD73 expression was not associated with clinical outcomes or PD-L1 expression in our cohorts, its contribution to immunosuppression and endometrial carcinoma progression may be context-dependent, restricted to specific molecular subsets or non–PD-L1–driven events. Its impact may also be less pronounced than that of cancer cell CD73 loss on disease progression.

The expression of mutant β-catenin in endometrial cancer cells provided a clinically relevant context for investigating how CD73 loss promotes a dual inflammatory and immunosuppressive microenvironment. Mechanistically, the induction of DKK1 by cancer cell CD73 loss is supported by our recent study. In *CTNNB1*-mutant endometrial cancer cells, we demonstrated that CD73 restrains mutant β-catenin at cell–cell adhesions (17). Loss of CD73 disrupts the membrane-associated pool of mutant β-catenin, increasing its nuclear translocation and transcriptional activity (17). Although our data here suggest that cancer cell CD73 loss may promote inflammation via DKK1-mediated stromal cell production of inflammatory mediators, this proposed mechanism remains indirect. We also note IL6 and CXCL1, although they are conventionally pro-inflammatory mediators, they can also recruit and activate suppressive myeloid populations that limit effective antitumor immunity (69, 70). DKK1 also has context-dependent immunomodulatory functions that can limit antitumor immunity, including promoting myeloid-mediated immune suppression and reduced CD8^+^ T-cell and NK-cell activity (36, 37). Thus, DKK1 may directly promote both inflammatory and immunosuppressive functions in CD73-low tumors. Studies incorporating immune cells are necessary in the future to address this limitation. Overall, our study demonstrates that cancer cell loss of CD73 promotes a dual inflammatory and immunosuppressive microenvironment in low-grade EEC, revealing a context-specific role for CD73 in the immune landscape of human tumors.

## Supplementary Material

Supplementary Table S1 Clinicopathological features of immunohistochemistry cohort of MSS/ MMR-proficient grade 2 endometrioid endometrial carcinomas.

Supplementary Table S2 Cytokine-cytokine receptor interaction group genes upregulated in NT5E low G2 EECMSS.

Supplementary Table S3 Immunosuppressive genes with ≥ 3 positive correlations with proinflammatory genes of the cytokine-cytokine receptor interaction group in NT5E low tumors.

Supplementary Table S4 Neutrophil activation pathways genes upregulated in NT5E-/- HEC-1-A cells.

Supplementary Fig. S1 Whole slide analysis of CD73 IHC staining patterns in G2 EECMSS.

Supplementary Fig. S2 Classifications for CD73 IHC staining patterns in epithelial cancer cells of G2 EECMSS.

Supplementary Fig. S3 Classifications for CD73 IHC staining in stromal cells of G2 EECMSS.

Supplementary Fig. S4 Cancer and stromal cell area comparisons for individual G2 EECMSS.

Supplementary Fig. S5 Clinical features and PTEN expression in relation to CD73 staining patterns across individual EECMSS.

Supplementary Fig. S6 Strong stromal cell CD73 expression is not associated with clinical features or patient outcomes in EECMSS.

Supplementary Fig. S7 PD-L1 does not associate with increased CD3+ immune cell infiltration in G2 EECMSS with high to moderate cancer cell CD73.

Supplementary Fig. S8 PD-L1 does not associate with increased CD8+ immune cell infiltration in cancer cell CD73-high or -low G2 EECMSS.

Supplementary Fig. S9 NT5E expression by cell type.

Supplementary Fig. S10 Cluster 9 and Cluster 12 are marked by cilium organization and assembly and reproductive function, respectively.

Supplementary Fig. S11 Cluster 1 makers IFIT1-, IFIT2-, and IFIT3-positive and -negative cells differ only modestly by NT5E status.

Supplementary Fig. S12 Cancer cell CD73 loss positively correlates with stromal cell CD73 expression in exon 3 CTNNB1-mutant EC.

Supplementary Fig. S13 Nuclear β-catenin regions show increased TGFB2 expression and inverse association with NT5E in exon 3 CTNNB1-mutant EC.

## Data Availability

RNA-seq data from HEC-1-A cells have been deposited in the NCBI Sequence Read Archive under BioProject ID PRJNA1534587. All other data are available from the corresponding author upon request. This article does not report original code.
