# Supplementary material for: Cancer Cell CD73 Loss Promotes a Dual Inflammatory and Immunosuppressive Microenvironment in Endometrial Carcinoma
Source: Cancer Res Commun. 2026 Nov 5;6(11):2602–18. doi: 10.1158/2767-9764.CRC-26-0167 (PMC13735482; doi:10.1158/2767-9764.CRC-26-0167)
Supplement: Supplementary file 1 [file crc-26-0167_supplementary_fig.s10_suppsf10.pdf]

Supplementary Fig. S10

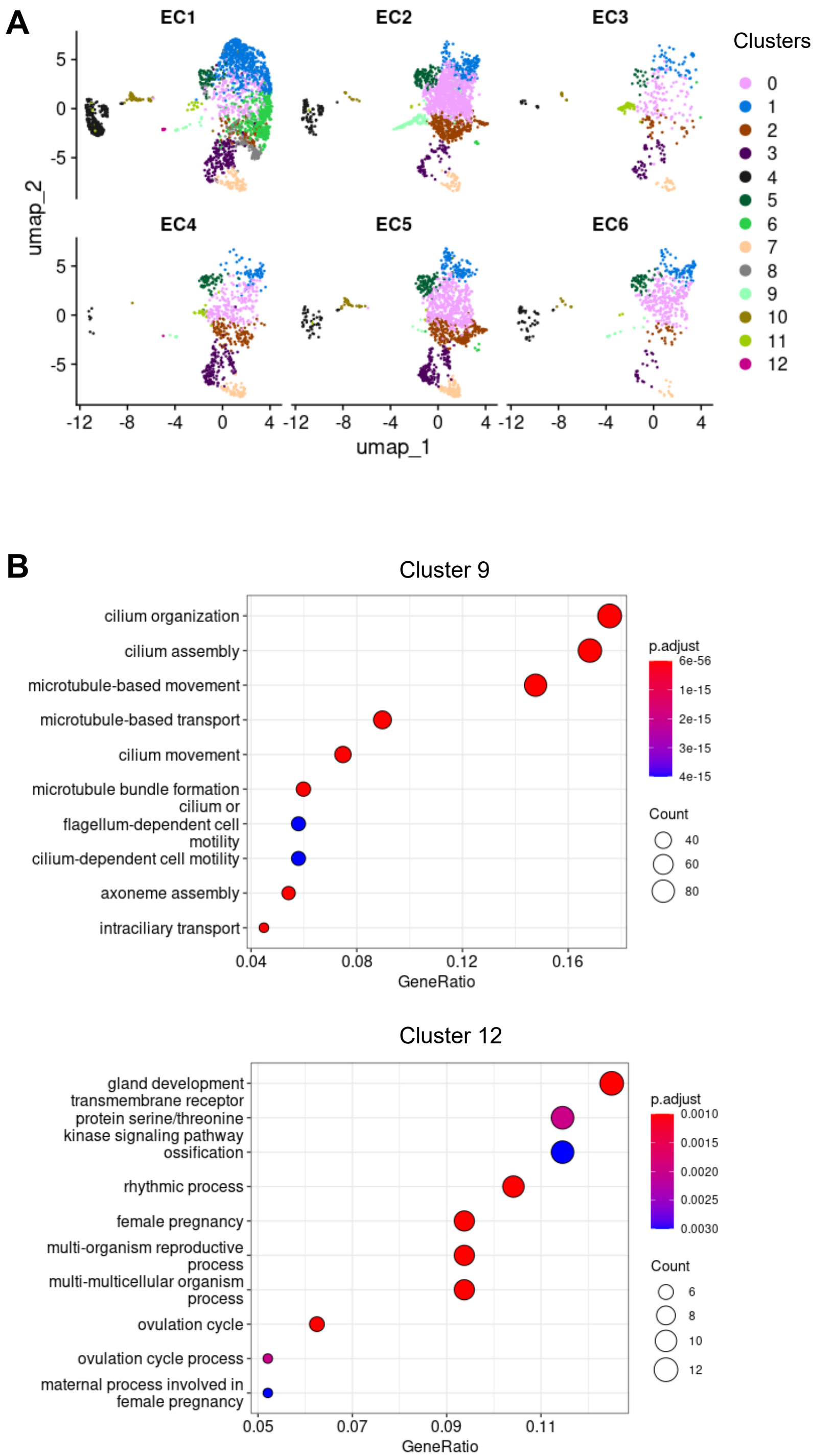

Supplementary Fig. S10. Cluster 9 and Cluster 12 are marked by cilium organization and assembly and reproductive function, respectively. (A) UMAP plots for individual tumors (EC1-EC6) showing the distribution of transcriptionally distinct clusters identified from the integrated single-cell RNAseq analysis in Fig. 6B. (B) Gene Ontology enrichment analysis for Cluster 9 and Cluster 12. Cluster 9 is enriched for cilium organization, cilium assembly, and microtubule-based movement, whereas Cluster 12 is enriched for reproductive and developmental processes. Clusters 9 and 12 represent the additional cell populations with relatively high *NT5E* expression and cell frequency identified in Fig. 7C.
