# Supplementary material for: Cancer Cell CD73 Loss Promotes a Dual Inflammatory and Immunosuppressive Microenvironment in Endometrial Carcinoma
Source: Cancer Res Commun. 2026 Nov 5;6(11):2602–18. doi: 10.1158/2767-9764.CRC-26-0167 (PMC13735482; doi:10.1158/2767-9764.CRC-26-0167)
Supplement: Supplementary file 2 [file crc-26-0167_supplementary_fig.s11_suppsf11.pdf]

Supplementary Fig. S11

A

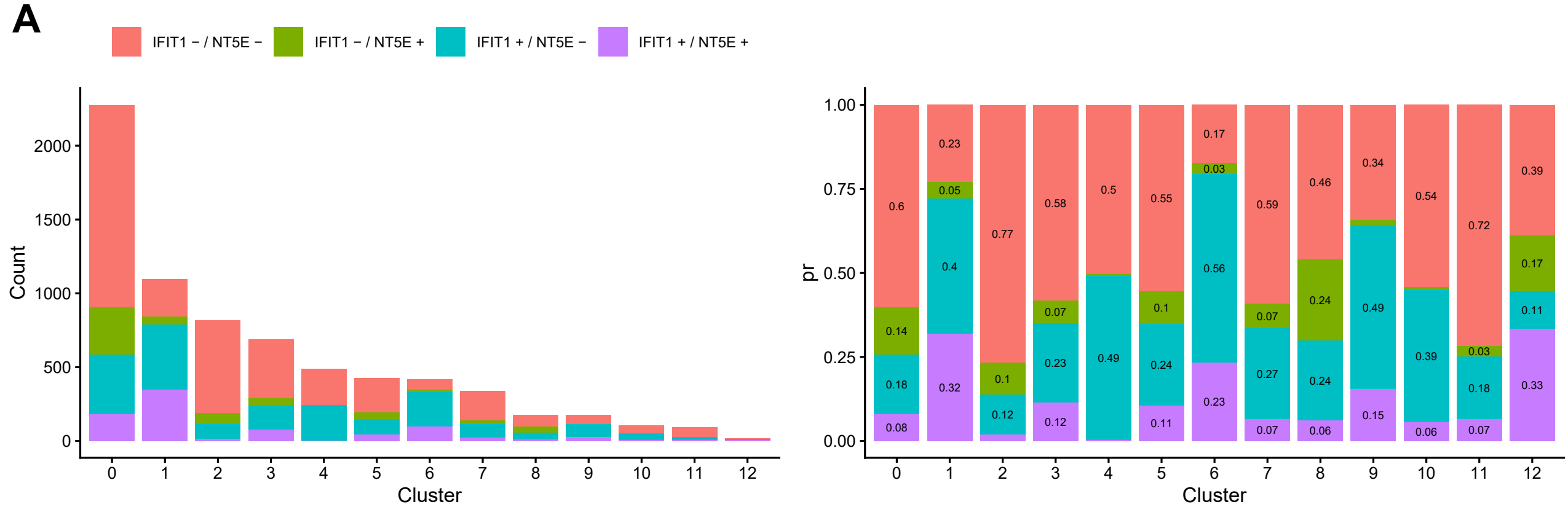

B

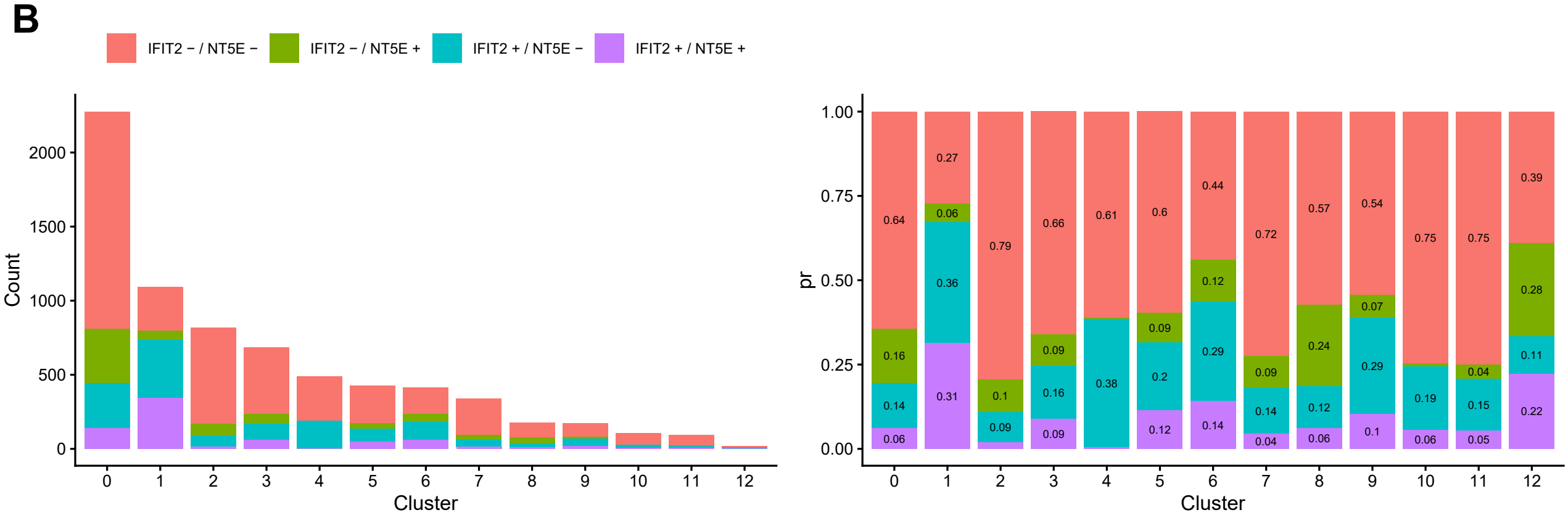

C

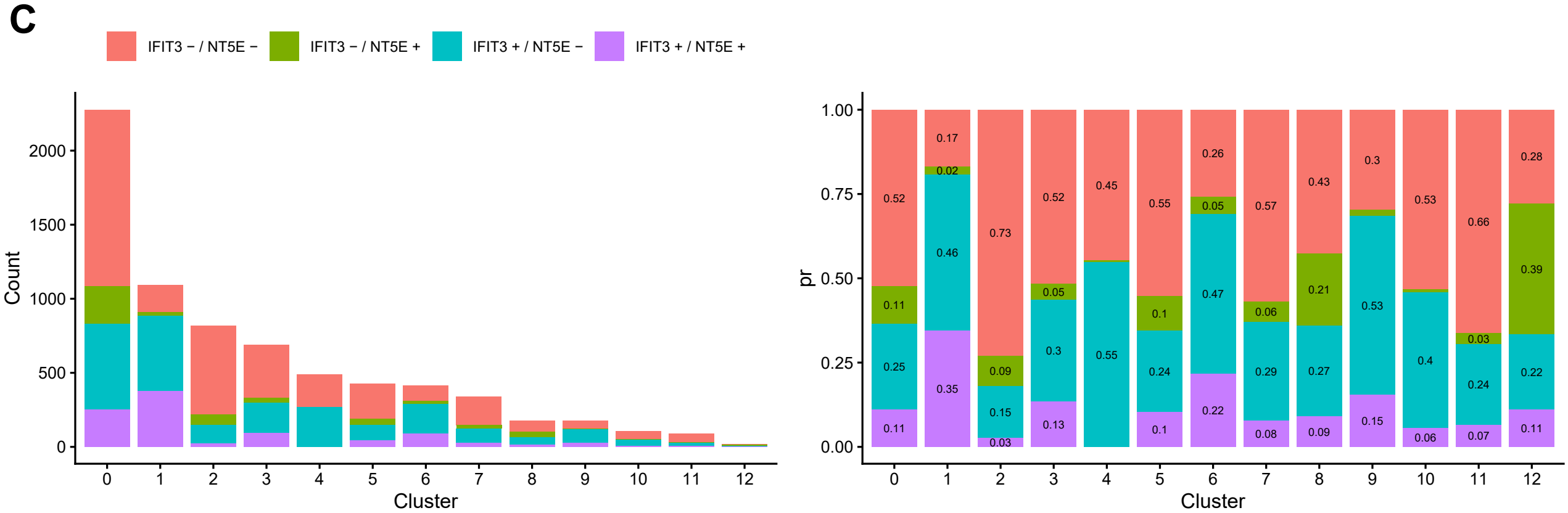

Supplementary Fig. S11. Cluster 1 makers *IFIT1-*, *IFIT2-*, and *IFIT3*-positive and -negative cells differ only modestly by *NT5E* status. Stacked bars are showing the distribution of *NT5E* and (A) *IFIT1*, (B) *IFIT2*, and (C) *IFIT3* positive and negative cells across clusters. Positive (+) expression for all genes was defined as values above 0; negative (-) expression was defined as 0. Stacked bars in the graph to the right show the proportion of *NT5E/CD274* expression groups within each cluster.
