# Supplementary material for: Cancer Cell CD73 Loss Promotes a Dual Inflammatory and Immunosuppressive Microenvironment in Endometrial Carcinoma
Source: Cancer Res Commun. 2026 Nov 5;6(11):2602–18. doi: 10.1158/2767-9764.CRC-26-0167 (PMC13735482; doi:10.1158/2767-9764.CRC-26-0167)
Supplement: Supplementary file 3 [file crc-26-0167_supplementary_fig.s12_suppsf12.pdf]

Supplementary Fig. S12

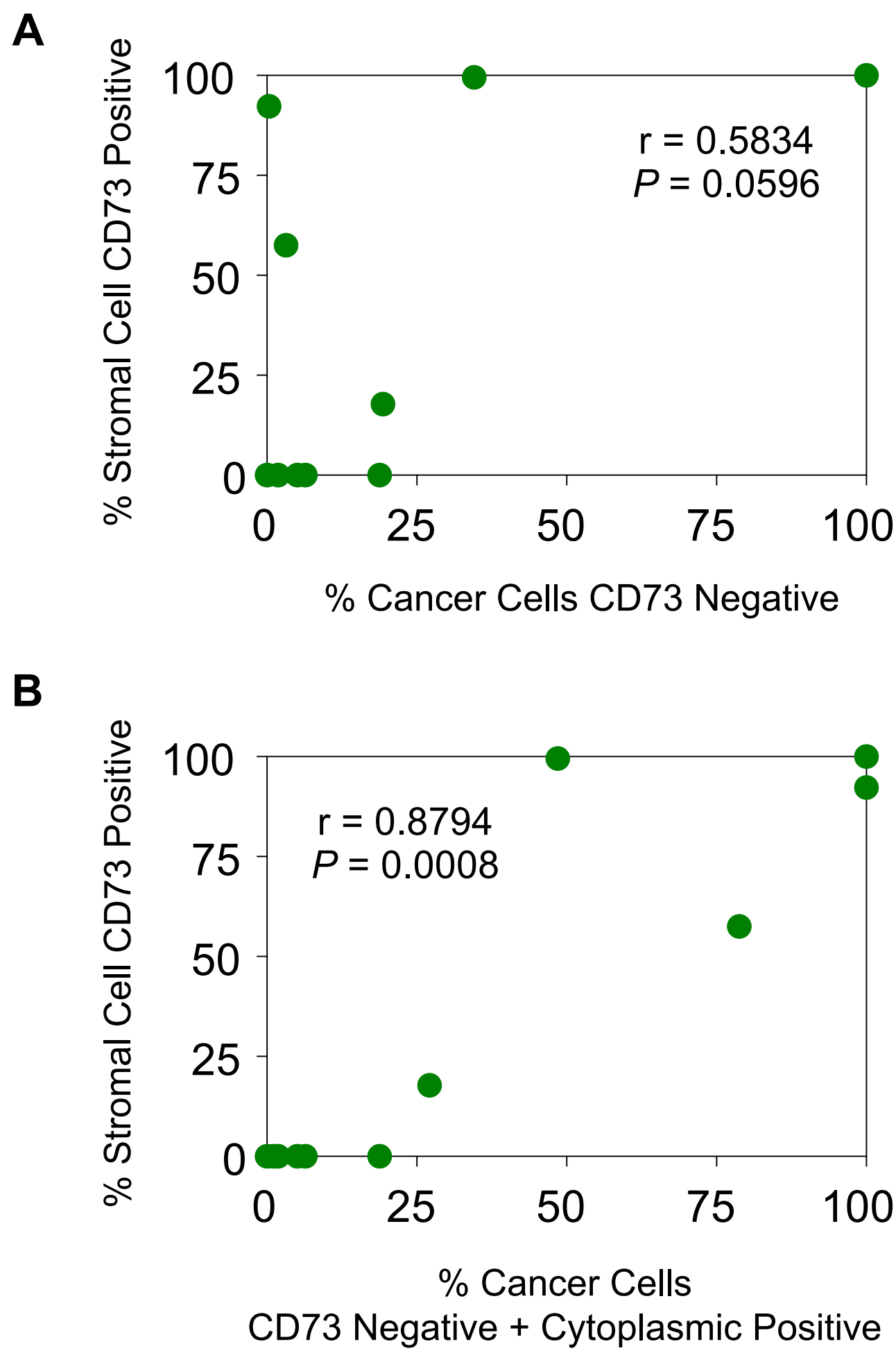

Supplementary Fig. S12. Cancer cell CD73 loss positively correlates with stromal cell CD73 expression in exon 3 *CTNNB1*-mutant EC. CD73 IHC staining was evaluated in 11 exon 3 *CTNNB1*-mutant EC. (A) Percent cancer cell area with complete loss of CD73 staining compared with percent stromal cell CD73-positive area. (B) Percent cancer cell area with loss of CD73 membrane localization, defined as CD73 Negative + Cytoplasmic Positive staining, compared with percent Stromal Cell CD73 Positive area. Tumors with greater cancer cell loss of CD73 membrane expression show increased stromal cell CD73 expression. (A-B) Spearman correlation.
