# Supplementary material for: Cancer Cell CD73 Loss Promotes a Dual Inflammatory and Immunosuppressive Microenvironment in Endometrial Carcinoma
Source: Cancer Res Commun. 2026 Nov 5;6(11):2602–18. doi: 10.1158/2767-9764.CRC-26-0167 (PMC13735482; doi:10.1158/2767-9764.CRC-26-0167)
Supplement: Supplementary file 4 [file crc-26-0167_supplementary_fig.s13_suppsf13.pdf]

Supplementary Fig. S13

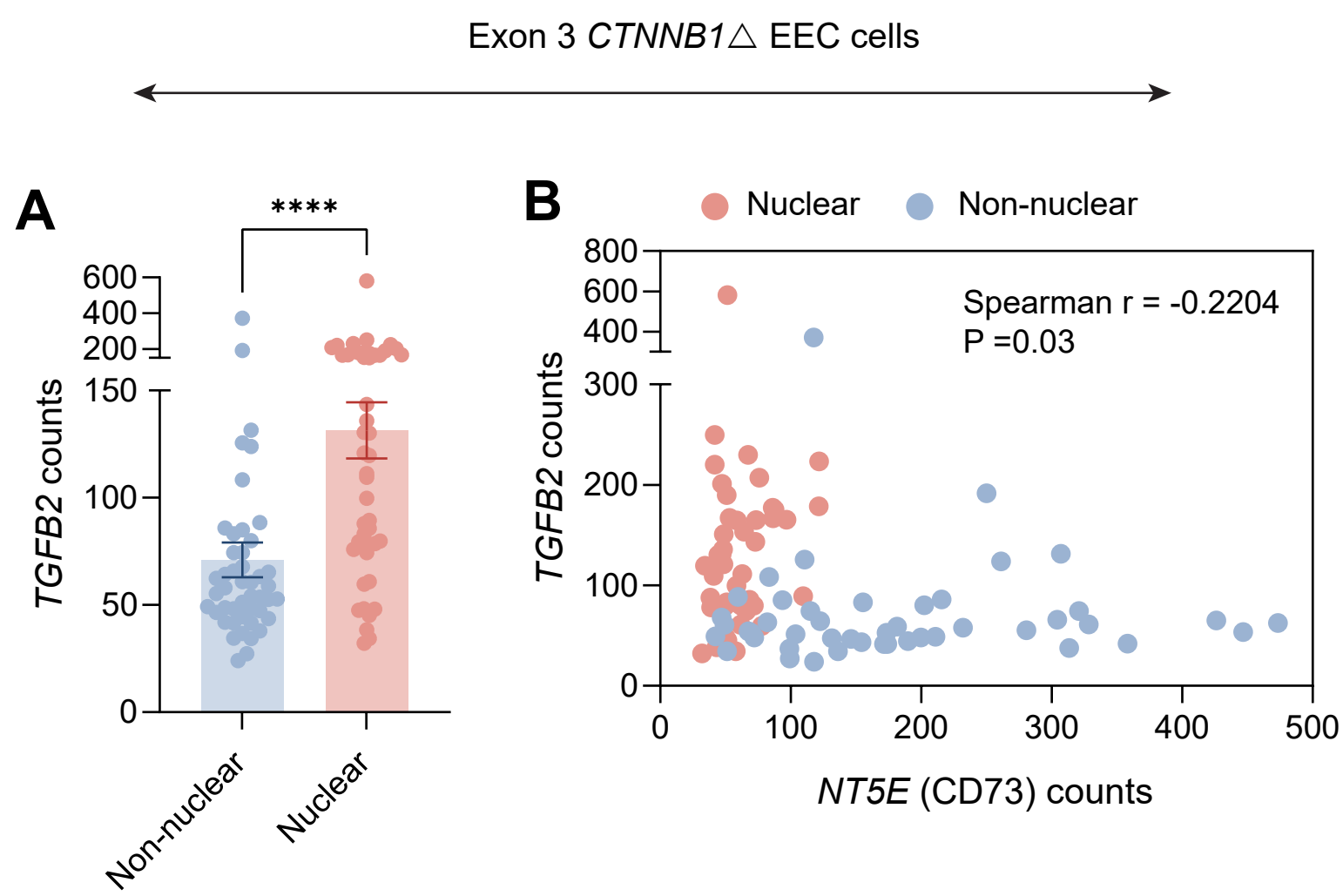

Supplementary Fig. S13. Nuclear β-catenin regions show increased *TGFB2* expression and inverse association with *NT5E* in exon 3 *CTNNB1*-mutant EC.

(A-B) GeoMx spatial profiling of *TGFB2* transcripts in nuclear and non-nuclear β-catenin regions of interest (ROIs) from 16 exon 3 *CTNNB1*-mutant ECs. (A) *TGFB2* transcript expression in 2-3 nuclear and non-nuclear β-catenin ROIs per individual tumor. (B) Inverse Spearman correlation between *TGFB2* and *NT5E* transcript expression across nuclear and non-nuclear β-catenin ROIs. Individual dots represent each ROI. Data are shown as mean ± SEM. (A) Mann–Whitney test. ****P < 0.0001.
