# Supplementary material for: Cancer Cell CD73 Loss Promotes a Dual Inflammatory and Immunosuppressive Microenvironment in Endometrial Carcinoma
Source: Cancer Res Commun. 2026 Nov 5;6(11):2602–18. doi: 10.1158/2767-9764.CRC-26-0167 (PMC13735482; doi:10.1158/2767-9764.CRC-26-0167)
Supplement: Supplementary file 5 [file crc-26-0167_supplementary_fig.s1_suppsf1.pdf]

Supplementary Fig. S1

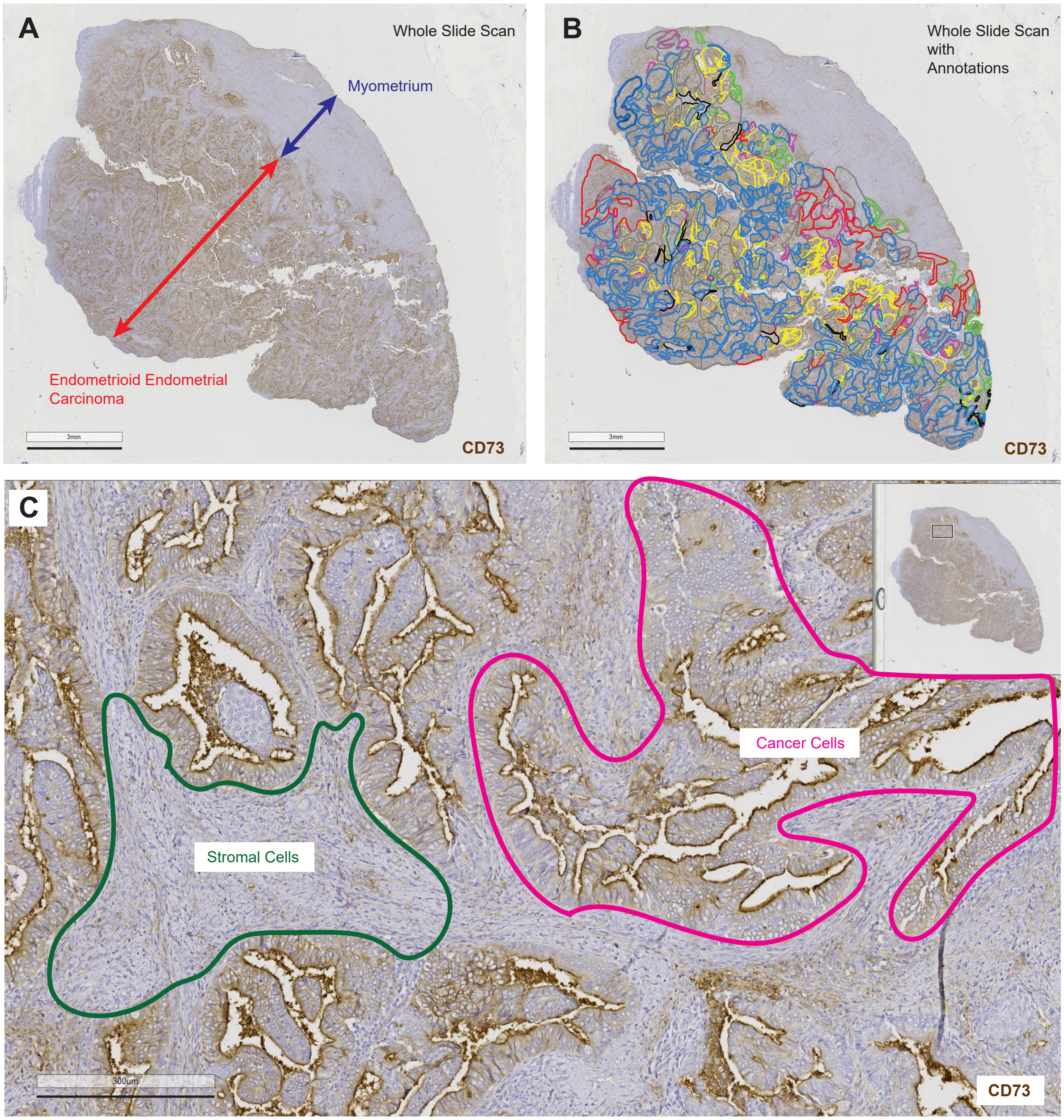

Supplementary Fig. S1. Whole slide analysis of CD73 IHC staining patterns in G2 EEC^{MSS}. (A) Whole slide view of a mismatch repair-intact grade 2 endometrioid endometrial carcinoma (G2 EEC^{MSS}) (scale bar: 3 mm) with immunohistochemistry (IHC) staining for CD73. (B) Whole slide view with annotations for distinct CD73 IHC staining patterns (classifications) in cancer cells and stromal cells. Annotations were made for ≥95% of the tumor area, with each colored outline corresponding to one assigned CD73 IHC staining classification (scale bar: 3 mm). Representative IHC images of each individual staining classification for cancer cells and stromal cells are shown in Supplementary Fig. S2 and S3. (C) Magnified view showing annotation boundaries used to distinguish cancer cell and stromal cell compartments (scale bar: 300 μm). The inset in the upper right corner indicates the location of the magnified region within the whole slide image.
