# Supplementary material for: Cancer Cell CD73 Loss Promotes a Dual Inflammatory and Immunosuppressive Microenvironment in Endometrial Carcinoma
Source: Cancer Res Commun. 2026 Nov 5;6(11):2602–18. doi: 10.1158/2767-9764.CRC-26-0167 (PMC13735482; doi:10.1158/2767-9764.CRC-26-0167)
Supplement: Supplementary file 6 [file crc-26-0167_supplementary_fig.s2_suppsf2.pdf]

Supplementary Fig. S2

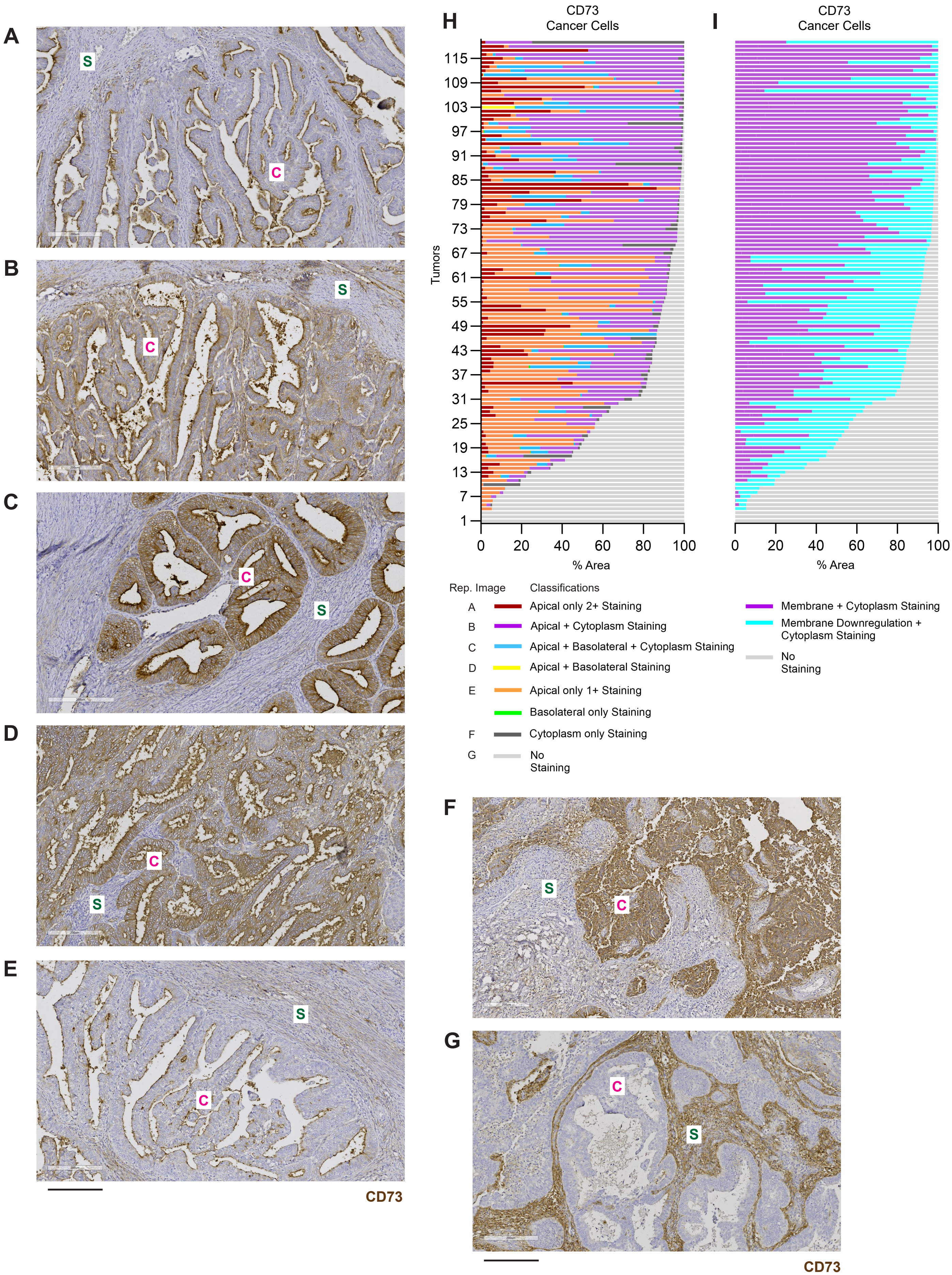

Supplementary Fig. S2. Classifications for CD73 IHC staining patterns in epithelial cancer cells of G2 EEC^{MSS}. Representative IHC images of cancer cell CD73 staining classifications: (A) Apical only 2+ Staining, defined as high CD73 expression restricted to the apical membrane; (B) Apical + Cytoplasm Staining, defined as CD73 staining on the apical membrane and in the cytoplasm; (C) Apical + Basolateral + Cytoplasm Staining, defined as CD73 staining on apical and basolateral membranes and in the cytoplasm; (D) Apical + Basolateral Staining, defined as CD73 staining on apical and basolateral membranes; (E) Apical only 1+ Staining, defined as low CD73 expression restricted to the apical membrane; (F) Cytoplasm only Staining, defined as CD73 staining in the cytoplasm; and (G) No Staining, defined as no CD73 staining in cancer cells. S indicates stromal cells; C indicates cancer cells. Scale bars: 200 μm. (H) Expression mapping of CD73 IHC cancer cell staining categories in 119 individual tumors; 8 staining classifications are shown. (I) Expression map of tumors with the 8 classifications of CD73 cancer cell staining shown in (H) simplified to 3 groups: Membrane + Cytoplasm Staining, includes the classifications Apical 2+ Staining, Apical + Basolateral Staining, Basolateral only Staining, Apical + Basolateral + Cytoplasm Staining, and Apical + Cytoplasm Staining from the original 8 classifications identified and shown in (H); Membrane Downregulation + Cytoplasmic Staining includes Cytoplasm only Staining and Apical only 1+ Staining classifications from the original 8 identified and shown in (H); No Staining indicates cancer cells with no CD73 staining. (H-I) Tumors are sorted by the percentage of cancer cell area with no CD73 staining.
