# Supplementary material for: Cancer Cell CD73 Loss Promotes a Dual Inflammatory and Immunosuppressive Microenvironment in Endometrial Carcinoma
Source: Cancer Res Commun. 2026 Nov 5;6(11):2602–18. doi: 10.1158/2767-9764.CRC-26-0167 (PMC13735482; doi:10.1158/2767-9764.CRC-26-0167)
Supplement: Supplementary file 7 [file crc-26-0167_supplementary_fig.s3_suppsf3.pdf]

Supplementary Fig. S3

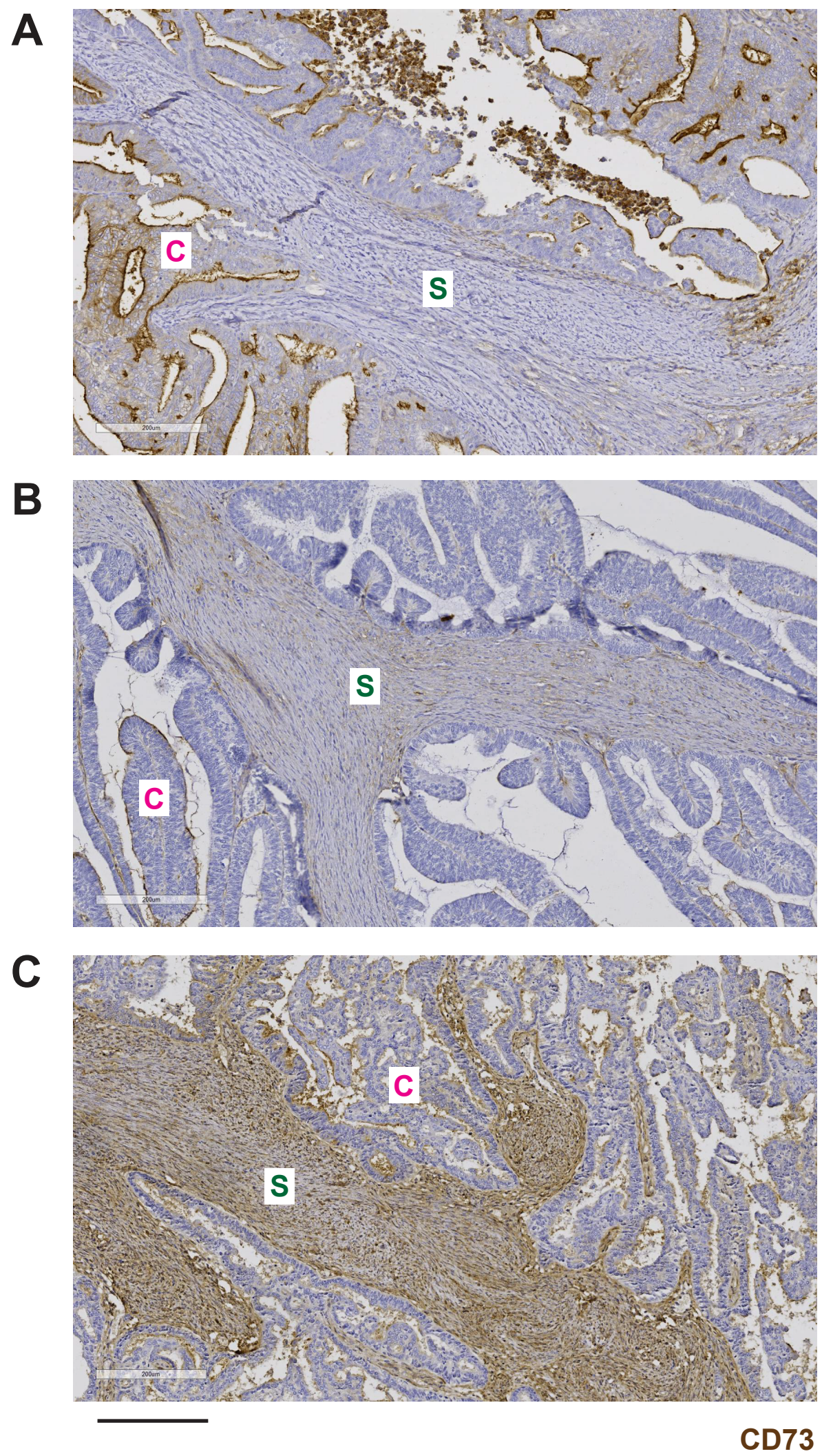

Supplementary Fig. S3. Classifications for CD73 IHC staining in stromal cells of G2 EEC^{MSS}. Representative IHC images of stromal CD73 IHC staining classifications: (A) No staining, defined as absence of CD73 staining in stromal cells; (B) Stromal Staining 1+, defined as low-level CD73 staining in stromal cells; and (C) Stromal Staining 2+, defined as high-level CD73 staining in stromal cells. S indicates stromal cells, whereas C indicates epithelial cancer cells. Scale bars, also shown by a single black bar, are 200 µm.
