# Supplementary material for: Cancer Cell CD73 Loss Promotes a Dual Inflammatory and Immunosuppressive Microenvironment in Endometrial Carcinoma
Source: Cancer Res Commun. 2026 Nov 5;6(11):2602–18. doi: 10.1158/2767-9764.CRC-26-0167 (PMC13735482; doi:10.1158/2767-9764.CRC-26-0167)
Supplement: Supplementary file 8 [file crc-26-0167_supplementary_fig.s4_suppsf4.pdf]

Supplementary Fig. S4

A

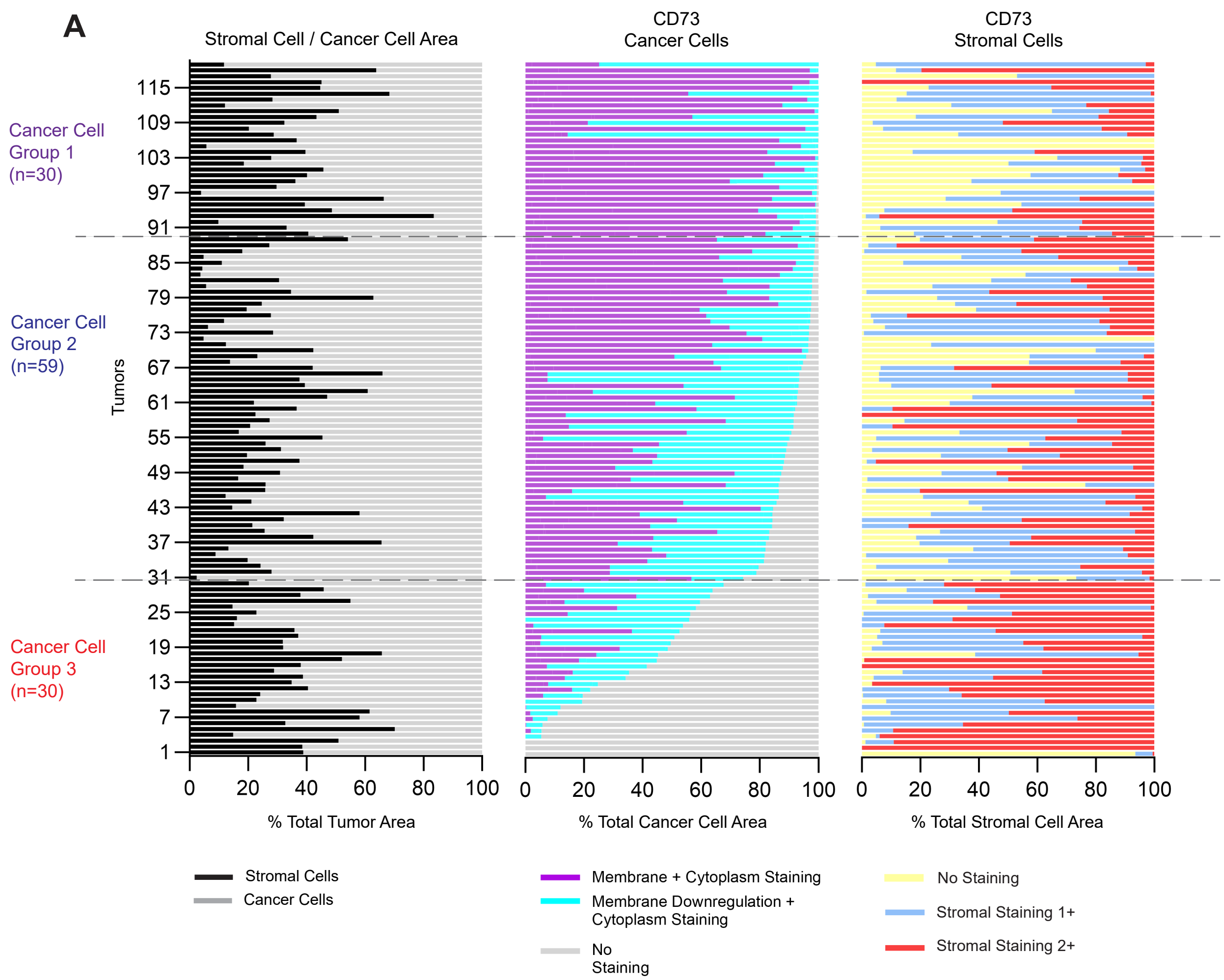

B

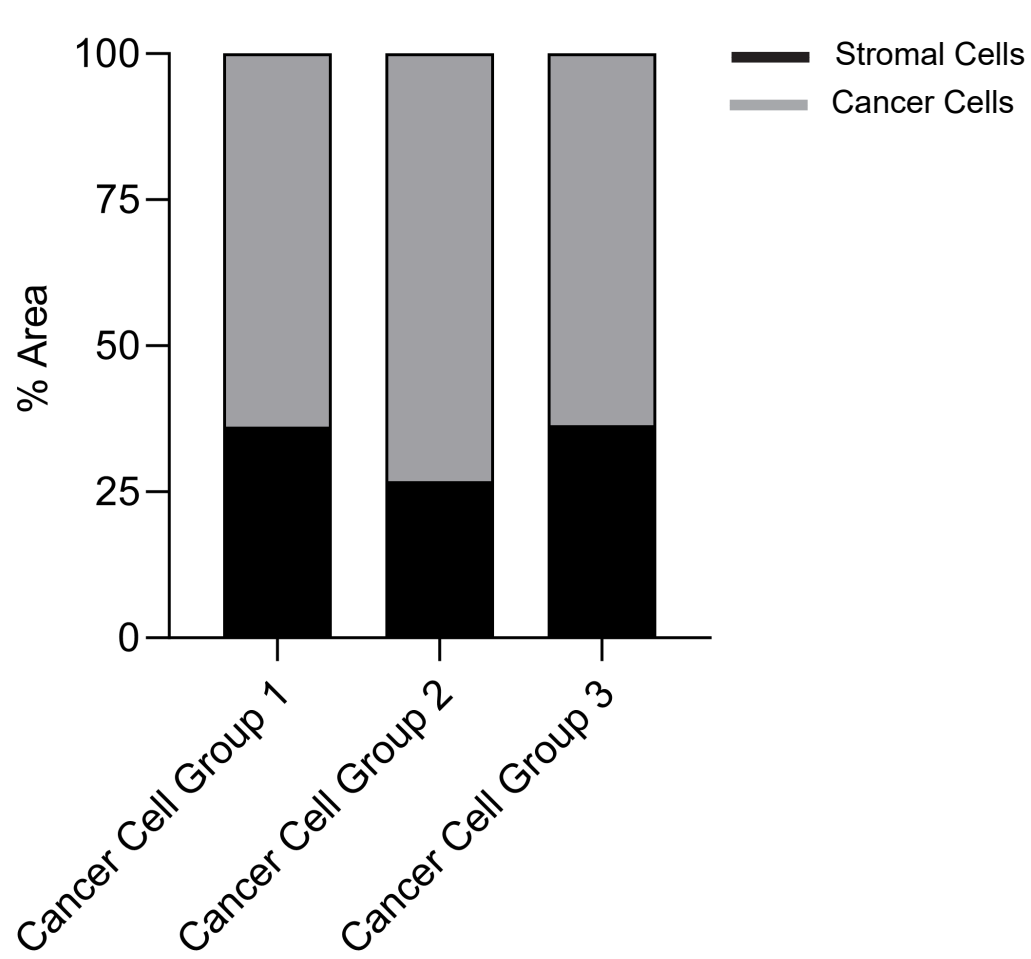

Supplementary Fig. S4. Cancer and stromal cell area comparisons for individual G2 EEC^{MSS}. (A) Relative proportions of cancer cell and stromal cell area for 119 individual tumors. Tumors are sorted by the percentage of cancer cell area with no CD73 staining. Corresponding CD73 staining classifications in cancer and stromal cell compartments are shown for comparison. Dotted lines indicate the upper and lower quartile cutoffs for cancer cell no staining for CD73, which define the three Cancer Cell Groups described in Fig. 1C. (B) Mean proportions of cancer cell and stromal cell area within each Cancer Cell Group.
