# Supplementary material for: Cancer Cell CD73 Loss Promotes a Dual Inflammatory and Immunosuppressive Microenvironment in Endometrial Carcinoma
Source: Cancer Res Commun. 2026 Nov 5;6(11):2602–18. doi: 10.1158/2767-9764.CRC-26-0167 (PMC13735482; doi:10.1158/2767-9764.CRC-26-0167)
Supplement: Supplementary file 9 [file crc-26-0167_supplementary_fig.s5_suppsf5.pdf]

Supplementary Fig. S5

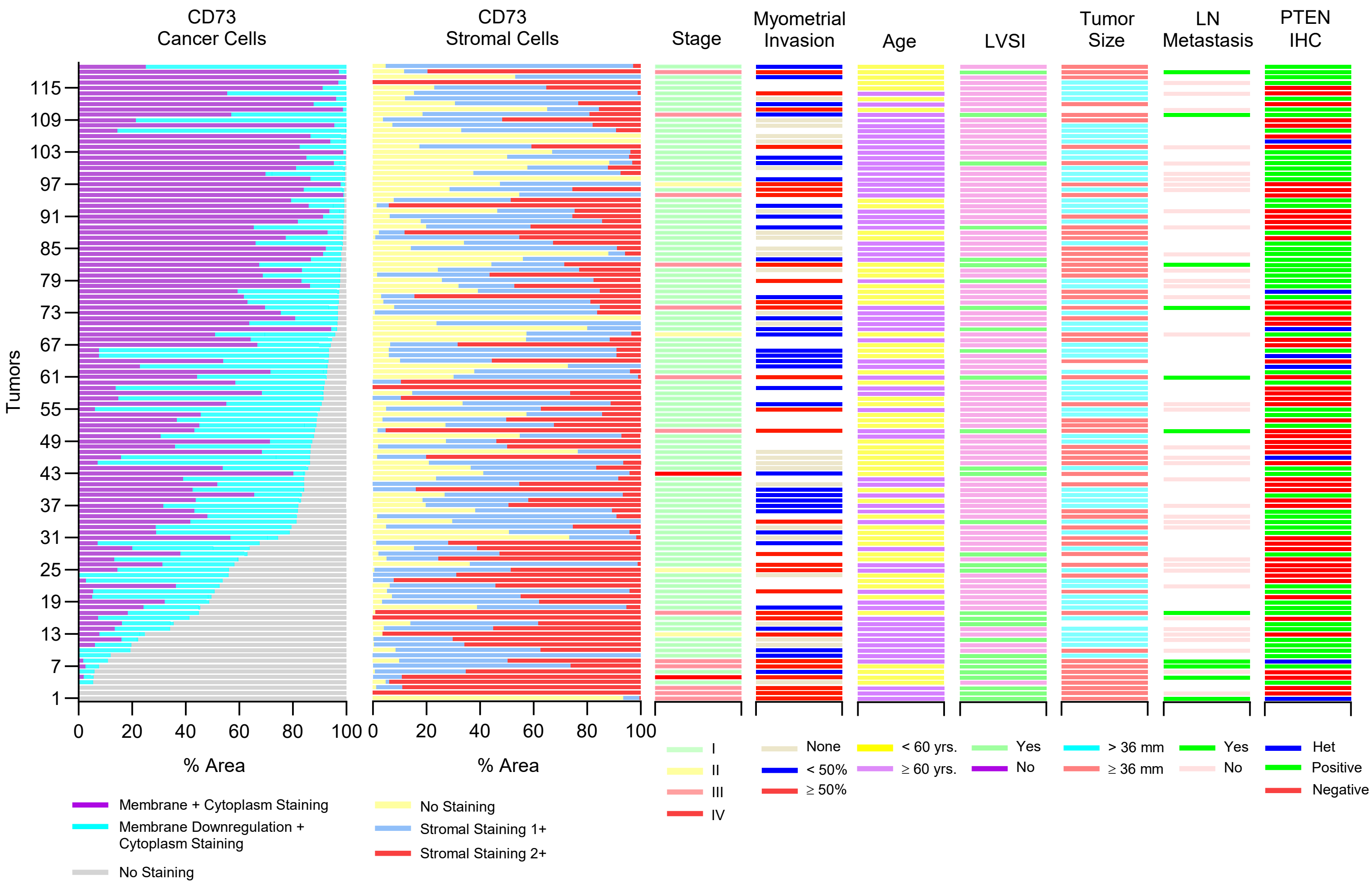

Supplementary Fig. S5. Clinical features and PTEN expression in relation to CD73 staining patterns across individual EEC^{MSS}. Individual 119 tumors are organized as described in Fig. 1. and Supplementary Fig. S2. CD73 staining classifications in cancer and stromal cells are shown alongside clinical features, including surgical stage, myometrial invasion, age, lymphovascular space invasion (LVSI), tumor size, and lymph node (LN) metastasis. PTEN immunohistochemistry (IHC) status is also shown. Het = heterogeneous PTEN staining. Although PTEN has been reported to have immunomodulatory actions (1), no association between CD73 staining patterns and PTEN status was observed in this study.

References

1. Cetintas VB, and Batada NN. Is there a causal link between PTEN deficient tumors and immunosuppressive tumor microenvironment? *J Transl Med.* 2020;18(1):45.
