# Supplementary material for: Cancer Cell CD73 Loss Promotes a Dual Inflammatory and Immunosuppressive Microenvironment in Endometrial Carcinoma
Source: Cancer Res Commun. 2026 Nov 5;6(11):2602–18. doi: 10.1158/2767-9764.CRC-26-0167 (PMC13735482; doi:10.1158/2767-9764.CRC-26-0167)
Supplement: Supplementary file 10 [file crc-26-0167_supplementary_fig.s6_suppsf6.pdf]

Supplementary Fig. S6

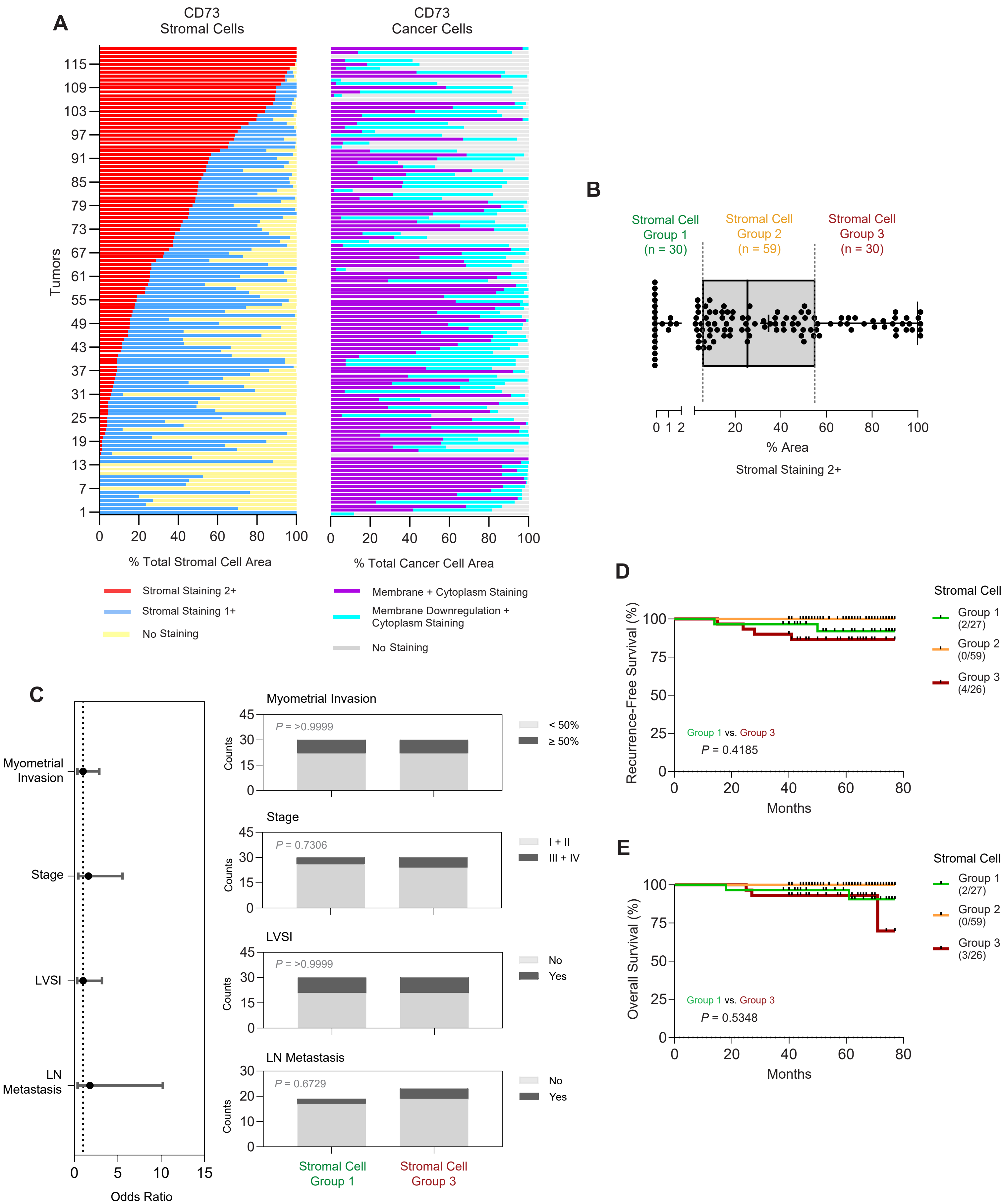

Supplementary Fig. S6. Strong stromal cell CD73 expression is not associated with clinical features or patient outcomes in EEC^{MSS}. (A) Expression mapping of CD73 IHC staining categories in stromal and cancer cells across 119 individual G2 EEC^{MSS}. Tumors are ordered by decreasing percentage of stromal cell area with CD73 Stromal Staining 2+. Corresponding cancer cell CD73 staining categories are shown for the same individual tumors for comparison. (B) Tumors were stratified into three groups based on quartiles of CD73 Stromal Staining 2+ area: Stromal Cell Group 1, low Stromal Staining 2+; Stromal Cell Group 2, intermediate Stromal Staining 2+; and Stromal Cell Group 3, high Stromal Staining 2+. The plot displays the distribution of tumors according to the percentage of stromal cell area with 2+ staining. Each point represents an individual tumor; the x-axis indicates the percentage of stromal cell area with 2+ staining, and the y-axis represents individual tumors ranked by Stromal Staining 2+ area. (C) Association of stromal CD73 expression with clinicopathologic features comparing Stromal Cell Group 1 and Stromal Cell Group 3. Features include myometrial invasion, stage, lymphovascular space invasion (LVSI), and lymph node (LN) metastasis. Bar plots show number of tumors. Fisher's exact test was used for group comparisons, and odds ratios were calculated using the Baptista-Pike method. (D-E) Recurrence-free survival (RFS) and overall survival (OS) comparisons in Stromal Cell Group 1 vs. Stromal Cell Group 3. Log-rank (Mantel-Cox) test; *P* values shown. (C-E) Information for clinical features, RFS, or OS was not available for all tumors.
