# Supplementary material for: Cancer Cell CD73 Loss Promotes a Dual Inflammatory and Immunosuppressive Microenvironment in Endometrial Carcinoma
Source: Cancer Res Commun. 2026 Nov 5;6(11):2602–18. doi: 10.1158/2767-9764.CRC-26-0167 (PMC13735482; doi:10.1158/2767-9764.CRC-26-0167)
Supplement: Supplementary file 11 [file crc-26-0167_supplementary_fig.s7_suppsf7.pdf]

Supplementary Fig. S7

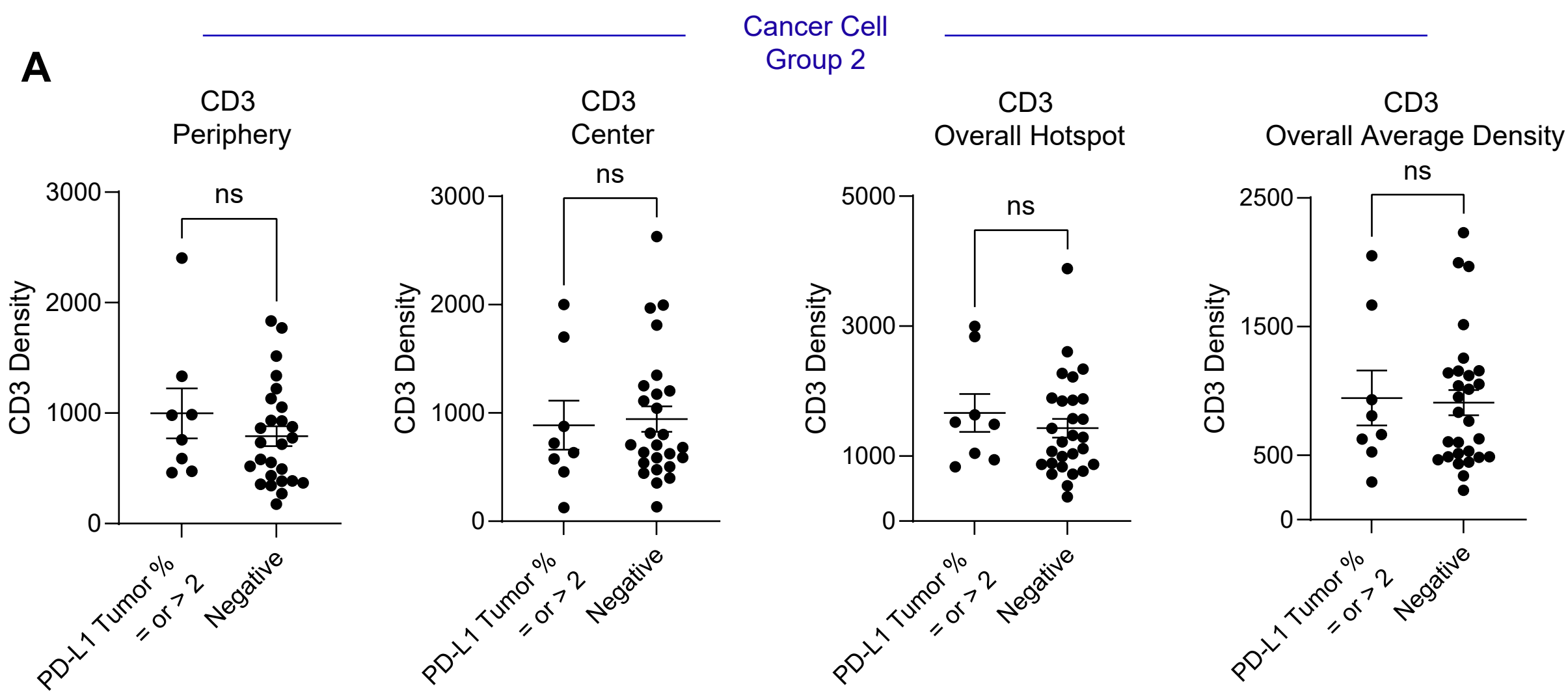

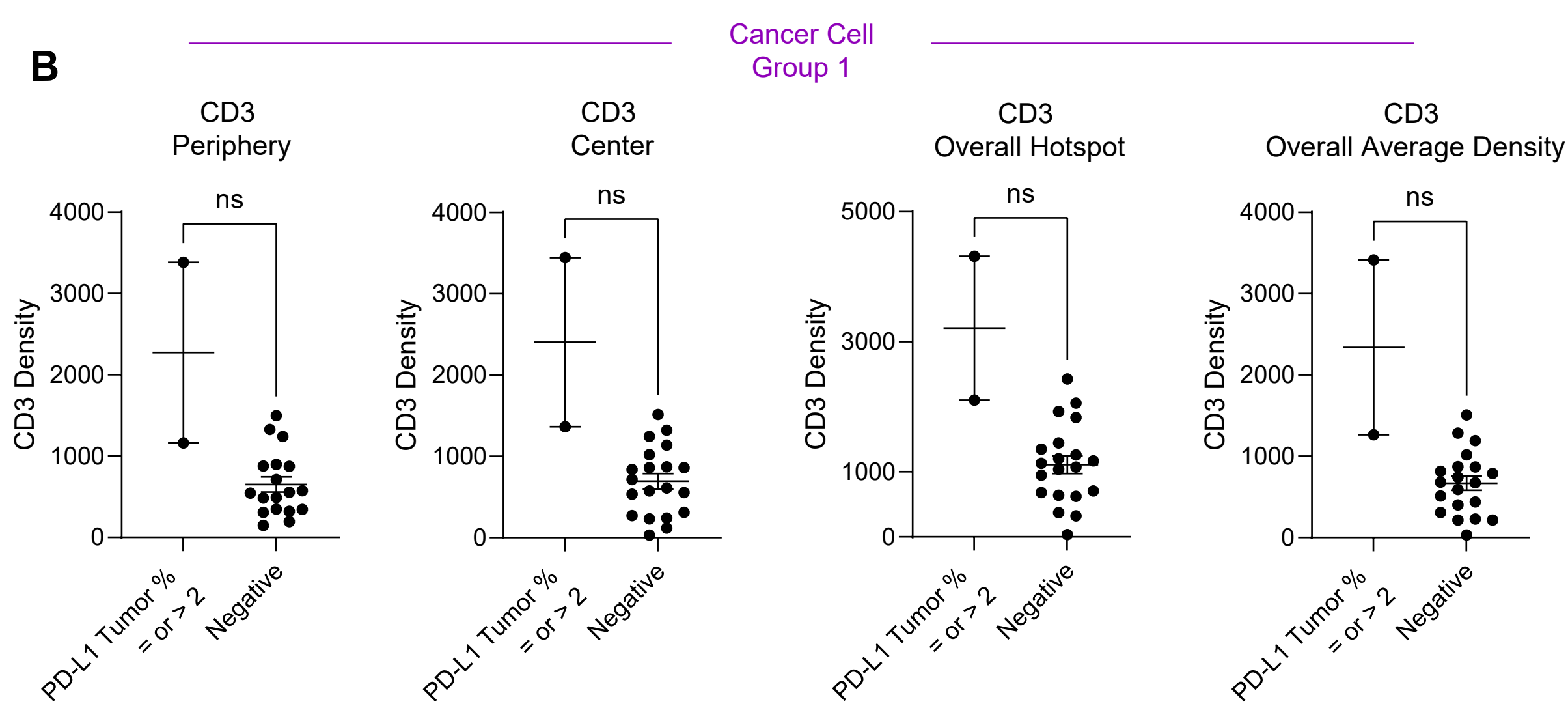

Supplementary Fig. S7. PD-L1 does not associate with increased CD3+ immune cell infiltration in G2 EEC^{MSS} with high to moderate cancer cell CD73. (A-B) CD3+ immune cell density in G2 EEC^{MSS} cases used in Fig. 1. Tumors were grouped according to PD-L1-positive tumor area (≥2% vs. negative). (A) Cancer Cell Group 2 includes tumors with intermediate loss of cancer cell CD73 protein expression, as shown in Fig. 1C. (B) Cancer Cell Group 1 includes tumors with ≤1.15% loss of cancer cell CD73 protein expression, as shown in Fig. 1C. CD3 density was assessed at the tumor periphery, tumor center, overall hotspot, and overall average density, as described by Crumley et al. (1). ns, not significant.

References

1. Crumley S, Kurnit K, Hudgens C, Fellman B, Tetzlaff MT, and Broaddus R. Identification of a subset of microsatellite-stable endometrial carcinoma with high PD-L1 and CD8+ lymphocytes. *Mod Pathol.* 2019;32(3):396-404.
