# Supplementary material for: Cancer Cell CD73 Loss Promotes a Dual Inflammatory and Immunosuppressive Microenvironment in Endometrial Carcinoma
Source: Cancer Res Commun. 2026 Nov 5;6(11):2602–18. doi: 10.1158/2767-9764.CRC-26-0167 (PMC13735482; doi:10.1158/2767-9764.CRC-26-0167)
Supplement: Supplementary file 12 [file crc-26-0167_supplementary_fig.s8_suppsf8.pdf]

Supplementary Fig. S8

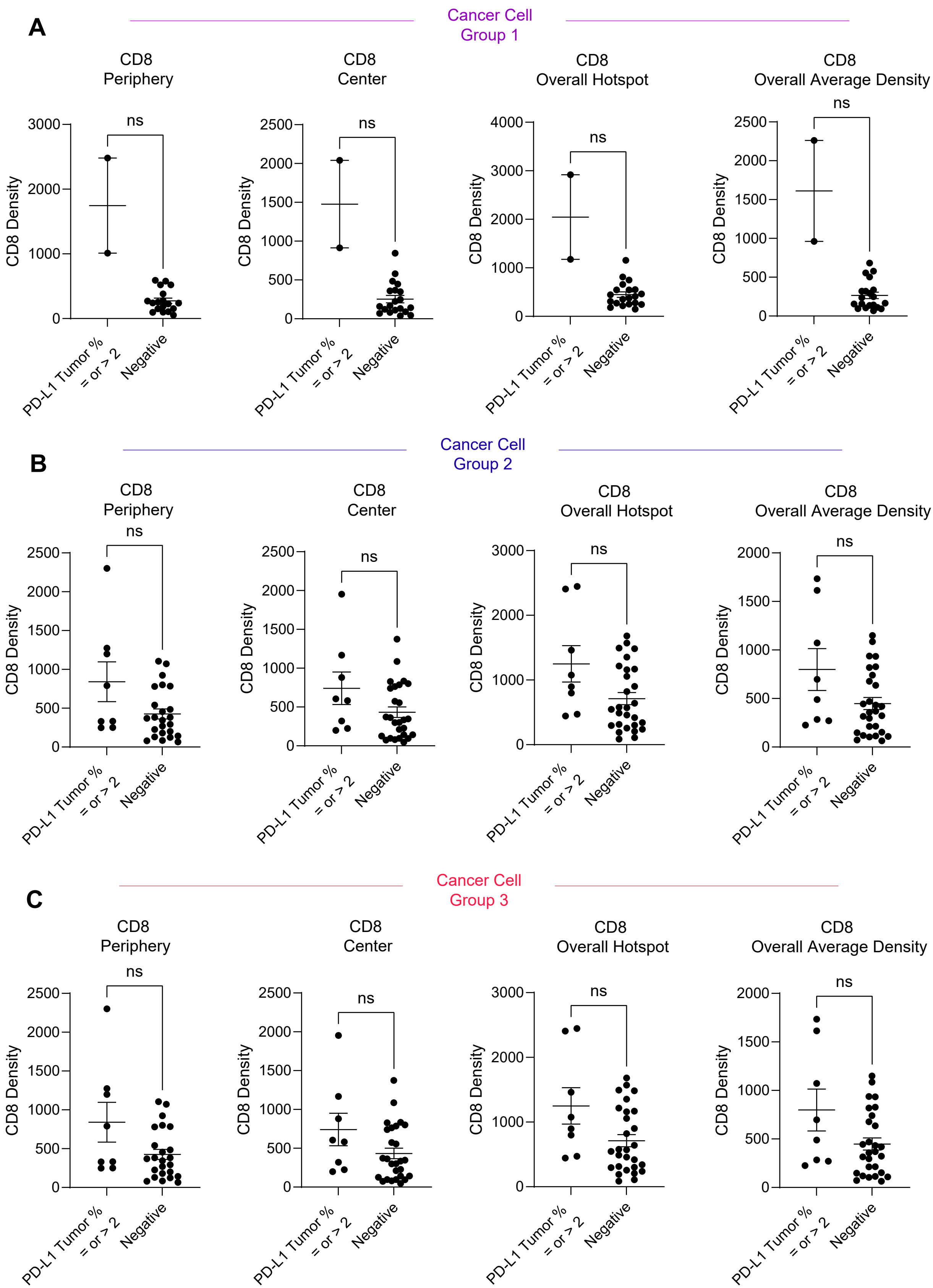

Supplementary Fig. S8. PD-L1 does not associate with increased CD8+ immune cell infiltration in cancer cell CD73-high or -low G2 EEC^{MSS}. (A-C) Tumor groups based on cancer cell CD73 expression that were defined in Fig. 1. (A) Cancer Cell Group 1 includes tumors with ≤1.15% loss of cancer cell CD73 protein expression. (B) Cancer Cell Group 2 includes tumors with intermediate loss of cancer cell CD73 protein expression. (C) Cancer Cell Group 3 includes tumors with high CD73 loss (32.38-100%). CD8 density was assessed at the tumor periphery, tumor center, overall hotspot, and overall average density, as described by Crumley et al. (1). ns, not significant.

References

1. Crumley S, Kurnit K, Hudgens C, Fellman B, Tetzlaff MT, and Broaddus R. Identification of a subset of microsatellite-stable endometrial carcinoma with high PD-L1 and CD8+ lymphocytes. *Mod Pathol.* 2019;32(3):396-404.
