# Supplementary material for: Cancer Cell CD73 Loss Promotes a Dual Inflammatory and Immunosuppressive Microenvironment in Endometrial Carcinoma
Source: Cancer Res Commun. 2026 Nov 5;6(11):2602–18. doi: 10.1158/2767-9764.CRC-26-0167 (PMC13735482; doi:10.1158/2767-9764.CRC-26-0167)
Supplement: Supplementary file 13 [file crc-26-0167_supplementary_fig.s9_suppsf9.pdf]

Supplementary Fig. S9

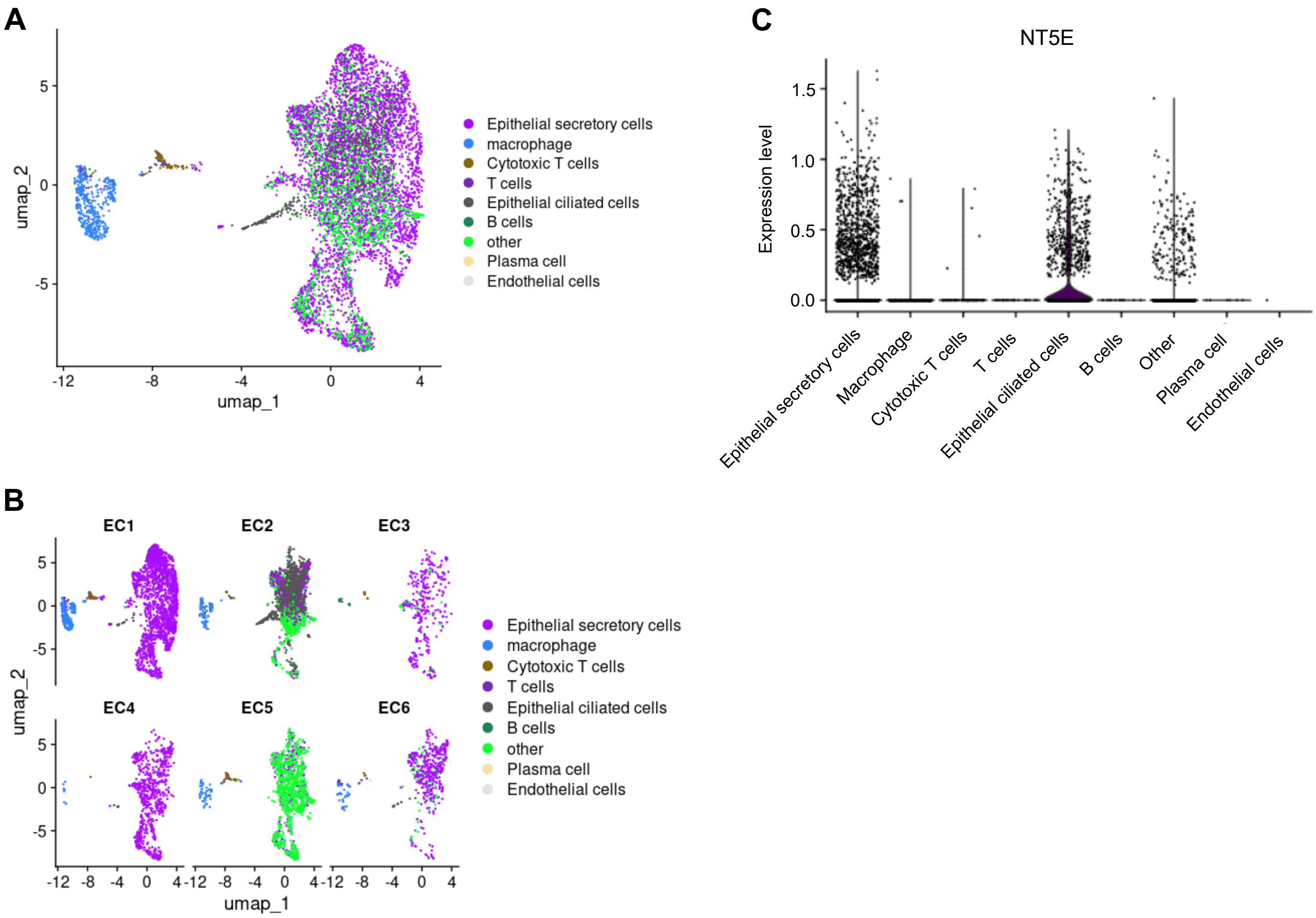

Supplementary Fig. S9. *NT5E* expression by cell type. Single-cell RNAseq of 6 EC, as previously described (1). (A) Integrated UMAP of single-cell RNAseq data from 6 EC, showing clustering of cells by transcriptional similarity and annotation into major cell populations (1). (B) UMAP plots for individual tumors EC1-EC6, showing the distribution of annotated cell types within each case. (C) *NT5E* expression across annotated cell types, showing predominant expression in epithelial secretory cells, with additional expression in epithelial ciliated cells and the "other" cell population.

References

1. Cochrane DR, Campbell KR, Greening K, Ho GC, Hopkins J, Bui M, et al. Single cell transcriptomes of normal endometrial derived organoids uncover novel cell type markers and cryptic differentiation of primary tumours. *J Pathol.* 2020;252(2):201-14.
