# Supplementary material for: Cancer Cell CD73 Loss Promotes a Dual Inflammatory and Immunosuppressive Microenvironment in Endometrial Carcinoma
Source: Cancer Res Commun. 2026 Nov 5;6(11):2602–18. doi: 10.1158/2767-9764.CRC-26-0167 (PMC13735482; doi:10.1158/2767-9764.CRC-26-0167)
Supplement: Supplementary file 14 [file crc-26-0167_supplementary_table_s1_suppst1.docx]

**Supplementary Table S1. Clinicopathological features of immunohistochemistry cohort of MSS/ MMR-proficient grade 2 endometrioid endometrial carcinomas.**

| **Characteristics (n = 119)** | **median (range)** |
| --- | --- |
| Age, years | 60 (27 – 82) |
| Tumor size, mm | 36 (16 –145) |
|  |  |
| FIGO Stage | **n (%)** |
| I | 101 (85) |
| II | 4 (3) |
| III | 7 (6) |
| IV | 7 (6) |
|  |  |
| Myometrial Invasion | **n (%)** |
| ≥ 50% | 27 (23) |
| < 50% | 33 (28) |
| No invasion | 28 (24) |
| Unknown | 31 (26) |
|  |  |
| Lymphovascular Space Invasion (LVSI) | **n (%)** |
| Yes | 30 (25) |
| No | 89 (75) |
|  |  |
| Metastasis to pelvic lymph nodes | **n (%)** |
| Yes | 11 (9) |
| No | 68 (57) |
| Lymph node dissection not performed | 40 (34) |
|  |  |
| Metastasis to para-aortic lymph nodes | **n (%)** |
| Yes | 5 (4) |
| No | 42 (35) |
| Lymph node dissection not performed | 72 (61) |
|  |  |
| PTEN immunohistochemistry | **n (%)** |
| Positive | 57 (48) |
| Heterogenous | 54 (45) |
| Negative | 8 (7) |
|  |  |
