# Supplementary material for: Cancer Cell CD73 Loss Promotes a Dual Inflammatory and Immunosuppressive Microenvironment in Endometrial Carcinoma
Source: Cancer Res Commun. 2026 Nov 5;6(11):2602–18. doi: 10.1158/2767-9764.CRC-26-0167 (PMC13735482; doi:10.1158/2767-9764.CRC-26-0167)
Supplement: Supplementary file 15 [file crc-26-0167_supplementary_table_s2_suppst2.docx]

**Supplementary Table S2. Cytokine-cytokine receptor interaction group genes upregulated in *NT5E* low G2 EEC^MSS^.**

| **Gene** | **Immune Function** | **Inflammatory**  **Response** | **Expression (cell type)** | **Roles in Cancer** | **References** |
| --- | --- | --- | --- | --- | --- |
| **CCL5**  *C-C Motif Chemokine Ligand 5* | Chemokine; attracts T cells, eosinophils, basophils, monocytes, NK cells, and dendritic cells to inflammatory sites | Proinflammatory | T cells, Th1 cells, monocytes, epithelial cancer cells, epithelial cells (endometrial) | Supports cancer cell growth, angiogenesis, metastasis; also supports the establishment of an immunosuppressive environment by recruiting immune cells which then gain tolerance | (1, 2) |
| **CCL8**  *C-C Motif Chemokine Ligand 8* | Chemokine; recruits and activates monocytes, T cells, and NK cells to sites of inflammation | Proinflammatory | Monocytes, Th2 cells, NK cells, epithelial cells, cancer epithelial cells (endometrial) | Has different effects on different tumors – both pro- and antitumor; can have antitumor effects by recruiting antitumor immune cells | (3, 4) |
| **CCL13**  *C-C Motif Chemokine Ligand 13* | Chemokine; recruits monocytes, lymphocytes, basophils | Proinflammatory | Monocytes, lymphocytes, basophils, epithelial cells, cancer epithelial cells | Enhances cancer cell proliferation and metastasis by recruiting immune cells that foster a supportive microenvironment for the tumor. | (5) |
| **CXCL9**  *C-X-C Motif Chemokine Ligand 9* | Chemokine; attracts Th1 cells and NK cells | Proinflammatory | Th1 cells, NK cells, epithelial cells, cancer epithelial cells (endometrial) | Inhibits tumor growth by recruiting Th1 cells and NK cells, key antitumor immune cells | (6) |
| **CXCL10**  *C-X-C Motif Chemokine Ligand 10* | Chemokine; attracts Th1 cells and NK cells | Proinflammatory | Monocytes, Th1 cells, NK cells, epithelial cells, cancer epithelial cells (endometrial) | Similar to CXCL9, inhibits tumor growth by attracting Th1 cells and NK cells. This chemokine enhances the antitumor immune response, leading to cancer cell elimination | (7) |
| **CXCL11**  *C-X-C Motif Chemokine Ligand 11* | Chemokine; attracts Th1 cells and NK cells | Proinflammatory | Th1 cells, NK cells, epithelial cells, cancer epithelial cells | Suppresses tumor growth by recruiting Th1 cells and NK cells, which exert strong antitumor immune responses. Helps to limit tumor proliferation and enhance immune surveillance against cancer cells | (8) |
| **CX3CL1**  *C-X3-C Motif Chemokine Ligand 1* | Chemokine; attracts monocytes, Th1 cells, and NK cells 🡪 involved in adhesion and migration | Proinflammatory | Monocytes, Th1 cells, NK cells, epithelial cells, cancer epithelial cells | Promotes tumor progression and metastasis by recruiting immune cells that can be co-opted by cancer cells. Its role in cell adhesion and migration helps facilitate the movement of cancer cells to other parts of the body, thus contributing to metastatic spread | (9) |
| **IL27RA** *Interleukin 27 Receptor Subunit Alpha* | Receptor for IL-27; involved in regulating T cell response | Proinflammatory and  Immuno-suppressive | T cells, including Th1, Th2, Tregs, epithelial cells, cancer epithelial cells (endometrial) | Modulates the immune response in the tumor microenvironment, with both proinflammatory and immunosuppressive effects. In certain cancers, it can support tumor immune evasion by promoting regulatory T cell (Treg) activity, while in others, it may enhance antitumor immune responses | (10) |
| **TNFRSF12A** *TNF Receptor Superfamily Member 12A* | Receptor for TWEAK; involved in cell survival, proliferation, and inflammation; enhances immune cell activation | Proinflammatory and  Immuno-suppressive | Epithelial cells, cancer epithelial cells (endometrial) | It contributes to immune cell activation; this activation if chronic can lead to inflammation which supports tumor progression and metastasis | (11) |
| **CD40** | Receptor involved in B cell activation, antigen-presenting cell activation; enhances Th1 and Th2 cell responses and modulates dendritic cell function | Proinflammatory and  Immuno-suppressive  Proinflammatory in colon epithelial cells and endometrium tissue | B cells, dendritic cells, T cells (Th1, Th2), epithelial cells, cancer epithelial cells (endometrial) | It can enhance antitumor immunity by activating antigen-presenting cells (APCs), but in some cancers, chronic activation of CD40 signaling can lead to immunosuppression and tumor growth, especially in certain epithelial cancers | (12-14) |
| **RELT**  *RELT TNF Receptor* | Receptor activates NF-κB pathway; involved in immune responses and lymphocyte activation; modulates Th1 and Th2 responses | Proinflammatory and  Immuno-suppressive | Lymphoid tissues, T cells (Th1, Th2), epithelial cells, cancer epithelial cells | Modulates immune responses in cancer, potentially promoting chronic inflammation that favors tumor progression. Activation of NF-κB pathway can drive immune responses that are permissive to tumor growth | (15) |
| **GDF15**  *Growth Differentiation Factor 15* | Growth differentiation factor; first identified as Macrophage inhibitor cytokine-1. Modulates inflammation and apoptosis; involved in regulating immune cell function and cytokine production | Proinflammatory and  Immuno-suppressive | Epithelial cells, cancer epithelial cells (endometrial) | Promotes tumor progression and metastasis by modulating immune cell function and inducing immune tolerance. It also influences cytokine production in the tumor microenvironment, promoting cancer cell survival and dissemination | (16, 17) |
| **INHA**  *Inhibin Subunit Alpha* | Hormone  Subunit of inhibin A complex, TGF-β family member associated with reproduction; associated with disease-associated intestinal inflammation in Crohn’s disease | Immuno-suppressive  (lung cancer) | Epithelial cells, cancer epithelial cells, Monocytes | Promotes an immunosuppressive microenvironment in certain cancers, such as lung cancer, by limiting immune responses against the tumor. However, it acts as a tumor suppressor in other contexts, such as in the gonads, adrenal glands, and prostate, where it helps to prevent cancer development | (18, 19) |
| **IL-32**  *Interleukin 32* | Cytokine;  promotes inflammation, enhances Th1 and Th2 cell responses and NK cell activity | Proinflammatory | T cells (Th1, Th2), NK cells, epithelial cells, epithelial cancer cells (endometrial) | Promotes tumor progression by enhancing proinflammatory cytokine production, which can lead to a tumor-promoting inflammatory environment. It also stimulates immune cells like Th1 and Th2 cells, which may support cancer cell survival and immune evasion | (20, 21) |
| **IL-11**  *Interleukin 11* | Cytokine; modulates hematopoiesis and inflammation; enhances stromal cell and immune cell interactions | Proinflammatory and  Immuno-suppressive | Stromal cells, epithelial cells, cancer epithelial cells (endometrial) | Promotes tumor growth and metastasis by modulating interactions between stromal cells and immune cells, leading to a supportive environment for tumor survival | (22) |

**References**

1.	Marques RE, Guabiraba R, Russo RC, and Teixeira MM. Targeting CCL5 in inflammation. *Expert Opin Ther Targets.* 2013;17(12):1439-60.

2.	Aldinucci D, Borghese C, and Casagrande N. The CCL5/CCR5 Axis in Cancer Progression. *Cancers (Basel).* 2020;12(7).

3.	Dangi A, Husain I, Jordan CZ, Yu S, Natesh N, Shen X, et al. Blocking CCL8-CCR8-Mediated Early Allograft Inflammation Improves Kidney Transplant Function. *J Am Soc Nephrol.* 2022;33(10):1876-90.

4.	Korbecki J, Kojder K, Siminska D, Bohatyrewicz R, Gutowska I, Chlubek D, et al. CC Chemokines in a Tumor: A Review of Pro-Cancer and Anti-Cancer Properties of the Ligands of Receptors CCR1, CCR2, CCR3, and CCR4. *Int J Mol Sci.* 2020;21(21).

5.	Li L, Dai F, Wang L, Sun Y, Mei L, Ran Y, et al. CCL13 and human diseases. *Front Immunol.* 2023;14:1176639.

6.	Neo SY, and Lundqvist A. The Multifaceted Roles of CXCL9 Within the Tumor Microenvironment. *Adv Exp Med Biol.* 2020;1231:45-51.

7.	Liu M, Guo S, and Stiles JK. The emerging role of CXCL10 in cancer (Review). *Oncol Lett.* 2011;2(4):583-9.

8.	Li Y, Han S, Wu B, Zhong C, Shi Y, Lv C, et al. CXCL11 Correlates with Immune Infiltration and Impacts Patient Immunotherapy Efficacy: A Pan-Cancer Analysis. *Front Immunol.* 2022;13:951247.

9.	Conroy MJ, and Lysaght J. CX3CL1 Signaling in the Tumor Microenvironment. *Adv Exp Med Biol.* 2020;1231:1-12.

10.	Zou J, Li Y, Liao N, Liu J, Zhang Q, Luo M, et al. Identification of key genes associated with polycystic ovary syndrome (PCOS) and ovarian cancer using an integrated bioinformatics analysis. *J Ovarian Res.* 2022;15(1):30.

11.	Wang D, Fung JN, Tuo Y, Hu L, and Chen C. TWEAK/Fn14 promotes apoptosis of human endometrial cancer cells via caspase pathway. *Cancer Lett.* 2010;294(1):91-100.

12.	Karnell JL, Rieder SA, Ettinger R, and Kolbeck R. Targeting the CD40-CD40L pathway in autoimmune diseases: Humoral immunity and beyond. *Adv Drug Deliv Rev.* 2019;141:92-103.

13.	Borcherding F, Nitschke M, Hundorfean G, Rupp J, von Smolinski D, Bieber K, et al. The CD40-CD40L pathway contributes to the proinflammatory function of intestinal epithelial cells in inflammatory bowel disease. *Am J Pathol.* 2010;176(4):1816-27.

14.	King AE, Kelly RW, Critchley HO, Malmstrom A, Sennstrom M, and Phipps RP. Cd40 expression in uterine tissues: a key regulator of cytokine expression by fibroblasts. *J Clin Endocrinol Metab.* 2001;86(1):405-12.

15.	Cusick JK, Alcaide J, and Shi Y. The RELT Family of Proteins: An Increasing Awareness of Their Importance for Cancer, the Immune System, and Development. *Biomedicines.* 2023;11(10).

16.	Joo M, Kim D, Lee MW, Lee HJ, and Kim JM. GDF15 Promotes Cell Growth, Migration, and Invasion in Gastric Cancer by Inducing STAT3 Activation. *Int J Mol Sci.* 2023;24(3).

17.	Siddiqui JA, Pothuraju R, Khan P, Sharma G, Muniyan S, Seshacharyulu P, et al. Pathophysiological role of growth differentiation factor 15 (GDF15) in obesity, cancer, and cachexia. *Cytokine Growth Factor Rev.* 2022;64:71-83.

18.	Singh P, Jenkins LM, Horst B, Alers V, Pradhan S, Kaur P, et al. Inhibin Is a Novel Paracrine Factor for Tumor Angiogenesis and Metastasis. *Cancer Res.* 2018;78(11):2978-89.

19.	Balanathan P, Ball EM, Wang H, Harris SE, Shelling AN, and Risbridger GP. Epigenetic regulation of inhibin alpha-subunit gene in prostate cancer cell lines. *J Mol Endocrinol.* 2004;32(1):55-67.

20.	Yu X, Zhou B, Zhang Z, Gao Q, Wang Y, Song Y, et al. Significant association between IL-32 gene polymorphisms and susceptibility to endometrial cancer in Chinese Han women. *Tumour Biol.* 2015;36(7):5265-72.

21.	Meng D, Dong H, Wang C, Zang R, and Wang J. Role of interleukin‑32 in cancer progression (Review). *Oncol Lett.* 2024;27(2):54.

22.	Lay V, Yap J, Sonderegger S, and Dimitriadis E. Interleukin 11 regulates endometrial cancer cell adhesion and migration via STAT3. *Int J Oncol.* 2012;41(2):759-64.
