# Supplementary material for: Cancer Cell CD73 Loss Promotes a Dual Inflammatory and Immunosuppressive Microenvironment in Endometrial Carcinoma
Source: Cancer Res Commun. 2026 Nov 5;6(11):2602–18. doi: 10.1158/2767-9764.CRC-26-0167 (PMC13735482; doi:10.1158/2767-9764.CRC-26-0167)
Supplement: Supplementary file 16 [file crc-26-0167_supplementary_table_s3_suppst3.docx]

**Supplemental Table S3. Immunosuppressive genes with ≥ 3 positive correlations with proinflammatory genes of the cytokine-cytokine receptor interaction group in *NT5E* low tumors.**

| **Gene** | **Immune Function** | **Inflammatory**  **Response** | **Expression (cell type)** | **Role in cancer** | **References** |
| --- | --- | --- | --- | --- | --- |
| **CCR4**  *C-C Motif Chemokine Receptor 4* | Receptor for CCL17 and CCL22 chemokines, involved in involved in chemotaxis of immune cells | Immuno-  suppressive | T cells, regulatory T cells, Th2 cells, cancer epithelial cells (endometrial) | Overexpressed in T-cell lymphomas and other cancers, promoting tumor progression and aiding in immune evasion by recruiting regulatory T cells (Tregs) to the tumor microenvironment | (1, 2) |
| **CD200R1**  *CD200 Receptor 1* | Inhibitory receptor for the OX-2 membrane glycoprotein–attenuates immune responses and is implicated in immune tolerance | Immuno-  suppressive | Myeloid cells, T cells, B cells, cancer epithelial cells | Overexpression results in immune suppression, aiding in immune evasion by inhibiting anti-tumor responses within the tumor microenvironment | (3, 4) |
| **CD28** | Co-stimulatory receptor for T cell activation and survival | Proinflammatory and  Immuno-suppressive | T cells, cancer epithelial cells | Plays a crucial role in T cell activation and survival, with its increased expression correlating with enhanced tumor growth, particularly in cancers like melanoma and lung cancer | (5) |
| **CD40LG**  *CD40 Ligand* | Ligand for CD40; involved in activation of antigen-presenting cells (e.g., macrophages, dendritic cells) | Proinflammatory and  Immuno-suppressive | T cells, activated CD4+ T cells, cancer epithelial cells | Mutations and dysregulation of CD40LG are implicated in lymphomas and other malignancies, where it contributes to abnormal immune activation and tumor growth | (6) |
| **CTLA4**  *Cytotoxic T-Lymphocyte Associated Protein 4* | Immune checkpoint inhibitor; downregulates immune responses by inhibiting T cell activation | Immuno-  suppressive | T cells, regulatory T cells, cancer epithelial cells (endometrial) | Overexpressed in numerous cancers, allowing tumors to evade immune detection by suppressing T cell activity and promoting immune tolerance | (7) |
| **FOXP3**  *Forkhead Box P3* | Transcription factor essential for regulatory T cell development | Immuno-suppressive | Regulatory T cells, cancer epithelial cells (endometrial) | Overexpression is associated with increased numbers of regulatory T cells, leading to immune suppression and poor prognosis in cancers such as endometrial cancer | (8) |
| **HLA-DRB1**  *Major Histocompatibility Complex, Class II, DR Beta 1* | Part of the MHC class II molecule, presents antigens to T cells | Proinflammatory | APCs, cancer epithelial cells | Altered expression disrupts antigen presentation, leading to immune evasion and contributing to the development of malignancies such as lymphomas | (9, 10) |
| **HLA-G**  *Major Histocompatibility Complex, Class I, G* | Immune checkpoint molecule; promotes immune tolerance by inhibiting NK cells and T cells | Immuno-  suppressive | Various tissues (saliva, ascitic fluid, plasma, thymus, seminal plasma, cerebrospinal fluid, and placenta), cancer epithelial cells | Frequently overexpressed in cancers, contributing to immune evasion by inhibiting immune surveillance mechanisms | (11) |
| **ICAM1**  *Intercellular Adhesion Molecule 1* | Cell adhesion molecule; facilitates leukocyte endothelial transmigration and recruitment of neutrophils | Proinflammatory | Endothelial cells, immune cells, cancer epithelial cells | Overexpression promotes tumor cell adhesion to endothelial cells, enhancing metastasis and cancer progression | (12, 13) |
| **IDO1**  *Indoleamine 2,3-Dioxygenase 1* | Enzyme involved in tryptophan metabolism; modulates immune responses by inhibiting T cell and NK cell function | Immuno-  suppressive | Dendritic cells, macrophages, tumor cells, cancer epithelial cells | Overexpressed in tumors, contributing to immune suppression and tolerance by depleting tryptophan, inhibiting T cell proliferation | (14) |
| **IFNG**  *Interferon Gamma* | Cytokine critical for innate and adaptive immunity; activates macrophages and enhances antigen presentation | Proinflammatory and  Immuno-suppressive | T cells, NK cells, cancer epithelial cells | Dysregulation can lead to either anti-tumor responses or immune evasion by upregulating immune checkpoints like PD-L1, depending on the tumor context | (15, 16) |
| **IL27**  *Interleukin 27* | Cytokine involved in regulating immune responses and inflammation; can activate NK cells and macrophages | Proinflammatory and  Immuno-suppressive | Dendritic cells, macrophages, cancer epithelial cells | Exhibits dual roles: promotes anti-tumor immune responses but can also support tumor growth depending on the tumor microenvironment | (17) |
| **CD274 (PD-L1)**  *Program Death-Ligand 1* | Immune checkpoint molecule; inhibits T cell activity and reduces macrophage activation | Immuno-  suppressive | Tumor cells, immune cells, cancer epithelial cells (endometrial) | Frequently overexpressed in many cancers, allowing tumors to evade immune detection and therapeutic interventions | (18, 19) |
| **IRAK3**  *Interleukin 1 Receptor Associated Kinase 3* | Negatively regulates Toll Like receptors; attenuates immune response and inhibits cytokine production | Immuno-  suppressive | Myeloid cells, macrophages, cancer epithelial cells | Overexpressed in tumors, contributing to immune suppression and tumor immune escape | (20) |
| **LGALS1**  *Galectin 1* | Encodes galectin-1; involved in cell-cell and cell-matrix interactions; can modulate neutrophil and macrophage function | Proinflammatory and  Immuno-suppressive | Various cell types, including immune and endothelial cells, cancer epithelial cells | Overexpression leads to tumor progression, immune evasion, and enhanced cancer cell survival | (21) |
| **LIF**  *LIF Interleukin 6 Family Cytokine* | Cytokine involved in cell differentiation, inflammation, and immune regulation; affects monocyte and macrophage function | Proinflammatory and  Immuno-suppressive | Various cell types, including immune cells, cancer epithelial cells | Overexpression drives tumor growth, immune suppression, and resistance to therapy in various cancers | (22) |
| **TNF**  *Tumor Necrosis Factor* | Cytokine part of acute phase reaction in systemic inflammation; activates neutrophils and macrophages | Proinflammatory and  Immuno-suppressive | Macrophages, T cells, NK cells, cancer epithelial cells (endometrial) | Dual role: promotes anti-tumor immune responses but also aids in tumor progression and immune evasion in certain contexts | (23) |
| **TNFRSF8**  *TNF Receptor Superfamily Member 8* | Receptor for TNF superfamily member 8. It’s involved in immune cell signaling and activation | Proinflammatory and  Immuno-suppressive | Various immune cells, especially in lymphoid tissues, cancer epithelial cells | Overexpressed in Hodgkin's lymphoma and other cancers, contributing to immune cell activation and tumor growth | (24) |
| **TNFSF9**  *TNF Superfamily Member 9* | Cytokine involved in T cell activation and survival; can enhance macrophage function | Proinflammatory and  Immuno-suppressive | Activated T cells, dendritic cells, cancer epithelial cells | Enhances antitumor immunity and may have therapeutic potential in cancer immunotherapy as it weakens the ability of CD8+ T cells to kill cancer cells | (25) |
| **VCAM1**  *Vascular Cell Adhesion Molecule 1* | Cell adhesion molecule involved in leukocyte-endothelial cell adhesion and signal transduction | Proinflammatory and  Immuno-suppressive | Endothelial cells, immune cells, cancer epithelial cells | Overexpression promotes tumor an inflammatory tumor microenvironment and immune evasion | (26, 27) |

Genes shown are immunosuppressive genes that were positively correlated with ≥ 3 significant correlations with cytokine-cytokine receptor interaction genes.

Genes are from the Harmonizome 3.0, Immunosuppression gene list.

**Supplemental Table 2 References**

1.	Yoshie O. CCR4 as a Therapeutic Target for Cancer Immunotherapy. *Cancers (Basel).* 2021;13(21).

2.	Bogacka J, Pawlik K, Ciapala K, Ciechanowska A, and Mika J. CC Chemokine Receptor 4 (CCR4) as a Possible New Target for Therapy. *Int J Mol Sci.* 2022;23(24).

3.	Vaine CA, and Soberman RJ. The CD200-CD200R1 inhibitory signaling pathway: immune regulation and host-pathogen interactions. *Adv Immunol.* 2014;121:191-211.

4.	Timmerman LM, de Graaf JF, Satravelas N, Kesmir C, Meyaard L, and van der Vlist M. Identification of a novel conserved signaling motif in CD200 receptor required for its inhibitory function. *PLoS One.* 2021;16(3):e0244770.

5.	Esensten JH, Helou YA, Chopra G, Weiss A, and Bluestone JA. CD28 Costimulation: From Mechanism to Therapy. *Immunity.* 2016;44(5):973-88.

6.	Marsman C, Verstegen NJ, Streutker M, Jorritsma T, Boon L, Ten Brinke A, et al. Termination of CD40L co-stimulation promotes human B cell differentiation into antibody-secreting cells. *Eur J Immunol.* 2022;52(10):1662-75.

7.	Buchbinder EI, and Desai A. CTLA-4 and PD-1 Pathways: Similarities, Differences, and Implications of Their Inhibition. *Am J Clin Oncol.* 2016;39(1):98-106.

8.	Mercer F, and Unutmaz D. The biology of FoxP3: a key player in immune suppression during infections, autoimmune diseases and cancer. *Adv Exp Med Biol.* 2009;665:47-59.

9.	Arango MT, Perricone C, Kivity S, Cipriano E, Ceccarelli F, Valesini G, et al. HLA-DRB1 the notorious gene in the mosaic of autoimmunity. *Immunol Res.* 2017;65(1):82-98.

10.	Hassan MM, Hussain MA, Ali SS, and Mahdi MA. In Silico Analysis: HLA-DRB1 Gene's Variants and Their Clinical Impact. *Cell Transplant.* 2023;32:9636897231184473.

11.	Zaborek-Lyczba M, Lyczba J, Mertowska P, Mertowski S, Hymos A, Podgajna M, et al. The HLA-G Immune Checkpoint Plays a Pivotal Role in the Regulation of Immune Response in Autoimmune Diseases. *Int J Mol Sci.* 2021;22(24).

12.	Qiu Z, Wang Y, Zhang Z, Qin R, Peng Y, Tang W, et al. Roles of intercellular cell adhesion molecule-1 (ICAM-1) in colorectal cancer: expression, functions, prognosis, tumorigenesis, polymorphisms and therapeutic implications. *Front Oncol.* 2022;12:1052672.

13.	Zhou Q, Xu J, Xu Y, Sun S, and Chen J. Role of ICAM1 in tumor immunity and prognosis of triple-negative breast cancer. *Front Immunol.* 2023;14:1176647.

14.	Zhai L, Ladomersky E, Lenzen A, Nguyen B, Patel R, Lauing KL, et al. IDO1 in cancer: a Gemini of immune checkpoints. *Cell Mol Immunol.* 2018;15(5):447-57.

15.	Mojic M, Takeda K, and Hayakawa Y. The Dark Side of IFN-gamma: Its Role in Promoting Cancer Immunoevasion. *Int J Mol Sci.* 2017;19(1).

16.	Alspach E, Lussier DM, and Schreiber RD. Interferon gamma and Its Important Roles in Promoting and Inhibiting Spontaneous and Therapeutic Cancer Immunity. *Cold Spring Harb Perspect Biol.* 2019;11(3).

17.	Fabbi M, Carbotti G, and Ferrini S. Dual Roles of IL-27 in Cancer Biology and Immunotherapy. *Mediators Inflamm.* 2017;2017:3958069.

18.	Gao G, Zhang XD, Qu H, Yao B, Zhou Y, Xiang J, et al. A comprehensive pan-cancer analysis of CD274 gene amplification, tumor mutation burden, microsatellite instability, and PD-L1 expression in Chinese cancer patients. *Ann Transl Med.* 2021;9(8):677.

19.	Mamat Yusof MN, Chew KT, Kampan N, Abd Aziz NH, Md Zin RR, Tan GC, et al. PD-L1 Expression in Endometrial Cancer and Its Association with Clinicopathological Features: A Systematic Review and Meta-Analysis. *Cancers (Basel).* 2022;14(16).

20.	Tunali G, Rubies Bedos M, Nagarajan D, Fridh P, Papakyriacou I, and Mao Y. IL-1 receptor-associated kinase-3 acts as an immune checkpoint in myeloid cells to limit cancer immunotherapy. *J Clin Invest.* 2023;133(7).

21.	Shi AP, Tang XY, Xiong YL, Zheng KF, Liu YJ, Shi XG, et al. Immune Checkpoint LAG3 and Its Ligand FGL1 in Cancer. *Front Immunol.* 2021;12:785091.

22.	Zhang C, Liu J, Wang J, Hu W, and Feng Z. The emerging role of leukemia inhibitory factor in cancer and therapy. *Pharmacol Ther.* 2021;221:107754.

23.	Wang X, and Lin Y. Tumor necrosis factor and cancer, buddies or foes? *Acta Pharmacol Sin.* 2008;29(11):1275-88.

24.	So T, and Ishii N. The TNF-TNFR Family of Co-signal Molecules. *Adv Exp Med Biol.* 2019;1189:53-84.

25.	Wu J, and Wang Y. Role of TNFSF9 bidirectional signal transduction in antitumor immunotherapy. *Eur J Pharmacol.* 2022;928:175097.

26.	Zhang S, Xie B, Wang L, Yang H, Zhang H, Chen Y, et al. Macrophage-mediated vascular permeability via VLA4/VCAM1 pathway dictates ascites development in ovarian cancer. *J Clin Invest.* 2021;131(3).

27.	Wu TC. The role of vascular cell adhesion molecule-1 in tumor immune evasion. *Cancer Res.* 2007;67(13):6003-6.
