# Supplementary material for: Cancer Cell CD73 Loss Promotes a Dual Inflammatory and Immunosuppressive Microenvironment in Endometrial Carcinoma
Source: Cancer Res Commun. 2026 Nov 5;6(11):2602–18. doi: 10.1158/2767-9764.CRC-26-0167 (PMC13735482; doi:10.1158/2767-9764.CRC-26-0167)
Supplement: Supplementary file 17 [file crc-26-0167_supplementary_table_s4_suppst4.docx]

**Supplemental Table S4. Neutrophil activation pathways genes upregulated in *NT5E^-/-^* HEC-1-A cells.**

| **Downregulated** | | | | |
| --- | --- | --- | --- | --- |
| **Gene** | **Functional Role** | **Inflammatory**  **Response** | **Expression (cell type)** | **References** |
| **GSTP1**  *Glutathione S- Transferase Pi 1* | Involved in detoxifying carcinogens and plays a role in regulating cell proliferation and apoptosis. | Immunosuppressive | Epithelial and cancer epithelial cells  (endometrial) | (1-4) |
| **PIGR**  *Polymeric Immunoglobulin Receptor* | Mediates the transcytosis of IgA antibodies, crucial in maintaining mucosal barriers against tumor-promoting agents | Immunosuppressive | Mucosal epithelial and mucosal cancer epithelial cells (endometrial) | (5-7) |
| **FOLR3**  *Folate Receptor Gamma* | Supports rapid cell division and growth typical of cancer cells by facilitating folate uptake | Unclear | Epithelial cells and cancer epithelial cells  (endometrial) | (8, 9) |
| **METTL7A** *Methyltransferase-like protein 7A* | Influences lipid metabolism which is often upregulated in cancer to support rapid cell growth | Immunosuppressive | Epithelial and cancer epithelial cells  (endometrial) | (10, 11) |
| **DOCK2**  *Dedicator Of Cytokinesis 2* | Modulates cell motility and invasion through actin cytoskeleton remodeling | Immunosuppressive | Hematopoietic cells | (12, 13) |
| **CD14** | Facilitates innate immune recognition which may be co-opted by cancer cells to evade immune surveillance | Immunosuppressive | Monocytes, macrophages, dendritic cells, cancer epithelial cells (endometrial) | (14, 15) |
|  | | | | |
| **Upregulated** | | | | |
| **Gene** | **Functional Role** | **Inflammatory Response** | **Expression (cell type)** | **References** |
| **CEACAM1**  *CEA Cell Adhesion Molecule 1* | Modulates cellular adhesion and signaling pathways that are often altered in cancer to promote survival and growth | Proinflammatory | Epithelial cells, immune cells, cancer epithelial cells (endometrial) | (16-18) |
| **ITCAM**  *Integrin Subunit Alpha M* | Facilitates interaction between tumor cells and the endothelium, promoting metastasis | Proinflammatory | Macrophages, endothelial cells, and cancer epithelial cells | (19, 20) |
| **HLA-C**  **Major** *Histocompatibility Complex, Class I, C* | Presents tumor antigens to cytotoxic T cells, integral to immune evasion strategies by cancer cells | Proinflammatory  and  Immuno-  suppressive | All nucleated cells (including endometrial cancer epithelial cells) | (21) |
| **HLA-B**  *Major Histocompatibility Complex, Class I, B* | Similar to HLA-C, facilitates antigen presentation, crucial for tumor immune surveillance or evasion | Proinflammatory  and  Immuno-  suppressive | All nucleated cells (including endometrial cancer epithelial cells) | (22) |
| **GSN**  *Gelsolin* | Regulates actin dynamics critical for cancer cell motility and invasion | Unclear | Epithelial cells, cancer epithelial cells  (endometrial) | (23) |
| **PLAUR**  *Plasminogen Activator, Urokinase Receptor* | Mediates proteolytic processes essential for cancer cell invasion and metastasis | Proinflammatory | Epithelial cells, cancer epithelial cells | (24) |
| **SIRPB1**  *Signal Regulatory Protein Beta 1* | Regulates signaling pathways that cancer cells may exploit to inhibit effective immune responses | Proinflammatory | Immune cells, dendritic cells, cancer epithelial cells | (25) |
| **CXCL1**  *C-X-C Motif Chemokine Ligand 1* | Attracts neutrophils to the tumor microenvironment, potentially supporting tumor growth and metastasis | Proinflammatory | Endothelial cells, fibroblasts, cancer epithelial cells (endometrial) | (26, 27) |
| **TMEM173**  *Stimulator of interferon response cGAMP interactor 1* | Activates pathways leading to antitumor immunity, potentially counteracted by cancer cells | Proinflammatory | Epithelial cells, cancer epithelial cells  (endometrial) | (28, 29) |
| **C3**  *Complement C3* | Part of the complement system which may be co-opted by cancers to promote immune evasion and tumor growth | Proinflammatory | Plasma, secreted by hepatocytes, also found in cancer epithelial cells (endometrial) | (30) |
| **CXCL8**  *C-X-C Motif Chemokine Ligand 8* | Promotes neutrophil recruitment and angiogenesis, both processes that can be utilized by tumors to grow and spread | Proinflammatory | Various cells, including endometrial cancer epithelial cells | (31) |
| **AOC1**  *Amine Oxidase Copper Containing 1* | Influences metabolism of polyamines necessary for rapid tumor cell proliferation | Unclear | Epithelial cells, cancer epithelial cells | (32) |
| **IQGAP2**  *IQ Motif Containing GTPase Activating Protein 2* | Modulates cell motility and morphology, often hijacked by cancer cells to facilitate invasion and metastasis | Unclear | Epithelial cells, cancer epithelial cells | (33) |
| **ITGAX**  *Integrin Subunit Alpha X* | Involved in adhesion and migration, critical for tumor-induced modulation of the immune response | Proinflammatory  and  Immuno-  suppressive | Immune cells | (34) |
| **PREX1**  *Phosphatidylinositol-3,4,5-Trisphosphate Dependent Rac Exchange Factor 1* | Activates signaling pathways that promote tumor cell growth, migration, and survival. | Unclear | Epithelial cells, cancer epithelial cells | (35, 36) |

Genes shown are those with ≥ 2 log2 Fold Change between NT5E^-/-^ vs. NT5E^+/+^ HEC-1-A cells.

.

**Supplemental Table 3 References**

1.	Zhao E, Hu K, and Zhao Y. Associations of the glutathione S-transferase P1 Ile105Val genetic polymorphism with gynecological cancer susceptibility: a meta-analysis. *Oncotarget.* 2017;8(25):41734-9.

2.	Ozerkan K, Atalay MA, Yakut T, Doster Y, Yilmaz E, and Karkucak M. Polymorphisms of glutathione-s-transferase M1, T1, and P1 genes in endometrial carcinoma. *Eur J Gynaecol Oncol.* 2013;34(1):42-7.

3.	Ogino S, Konishi H, Ichikawa D, Matsubara D, Shoda K, Arita T, et al. Glutathione S-transferase Pi 1 is a valuable predictor for cancer drug resistance in esophageal squamous cell carcinoma. *Cancer Sci.* 2019;110(2):795-804.

4.	Shishido Y, Tomoike F, Kuwata K, Fujikawa H, Sekido Y, Murakami-Tonami Y, et al. A Covalent Inhibitor for Glutathione S-Transferase Pi (GSTP(1-1) ) in Human Cells. *Chembiochem.* 2019;20(7):900-5.

5.	Chae J, Choi J, and Chung J. Polymeric immunoglobulin receptor (pIgR) in cancer. *J Cancer Res Clin Oncol.* 2023;149(19):17683-90.

6.	Tey SK, Wong SWK, Chan JYT, Mao X, Ng TH, Yeung CLS, et al. Patient pIgR-enriched extracellular vesicles drive cancer stemness, tumorigenesis and metastasis in hepatocellular carcinoma. *J Hepatol.* 2022;76(4):883-95.

7.	Zhou M, Liu C, Cao G, Gao H, and Zhang Z. Expression of polymeric immunoglobulin receptor and its biological function in endometrial adenocarcinoma. *J Cancer Res Ther.* 2019;15(2):420-5.

8.	Yuan Y, Nymoen DA, Dong HP, Bjorang O, Shih Ie M, Low PS, et al. Expression of the folate receptor genes FOLR1 and FOLR3 differentiates ovarian carcinoma from breast carcinoma and malignant mesothelioma in serous effusions. *Hum Pathol.* 2009;40(10):1453-60.

9.	Senol S, Ceyran AB, Aydin A, Zemheri E, Ozkanli S, Kosemetin D, et al. Folate receptor alpha expression and significance in endometrioid endometrium carcinoma and endometrial hyperplasia. *Int J Clin Exp Pathol.* 2015;8(5):5633-41.

10.	Liu Z, Chen Y, and Shen T. Evidence Based on an Integrative Analysis of Multi-Omics Data on METTL7A as a Molecular Marker in Pan-Cancer. *Biomolecules.* 2023;13(2).

11.	Lee TY, Martinez-Outschoorn UE, Schilder RJ, Kim CH, Richard SD, Rosenblum NG, et al. Metformin as a Therapeutic Target in Endometrial Cancers. *Front Oncol.* 2018;8:341.

12.	Guo X, and Chen SY. Dedicator of Cytokinesis 2 in Cell Signaling Regulation and Disease Development. *J Cell Physiol.* 2017;232(8):1931-40.

13.	Chen Y, Meng F, Wang B, He L, Liu Y, and Liu Z. Dock2 in the development of inflammation and cancer. *Eur J Immunol.* 2018;48(6):915-22.

14.	Cheah MT, Chen JY, Sahoo D, Contreras-Trujillo H, Volkmer AK, Scheeren FA, et al. CD14-expressing cancer cells establish the inflammatory and proliferative tumor microenvironment in bladder cancer. *Proc Natl Acad Sci U S A.* 2015;112(15):4725-30.

15.	Stenzel AE, Abrams SI, Joseph JM, Goode EL, Tario JD, Jr., Wallace PK, et al. Circulating CD14(+) HLA-DR(lo/-) monocytic cells as a biomarker for epithelial ovarian cancer progression. *Am J Reprod Immunol.* 2021;85(3):e13343.

16.	Thomas J, Klebanov A, John S, Miller LS, Vegesna A, Amdur RL, et al. CEACAMS 1, 5, and 6 in disease and cancer: interactions with pathogens. *Genes Cancer.* 2023;14:12-29.

17.	Dankner M, Gray-Owen SD, Huang YH, Blumberg RS, and Beauchemin N. CEACAM1 as a multi-purpose target for cancer immunotherapy. *Oncoimmunology.* 2017;6(7):e1328336.

18.	Bamberger AM, Briese J, Gotze J, Erdmann I, Schulte HM, Wagener C, et al. Stimulation of CEACAM1 expression by 12-O-tetradecanoylphorbol-13-acetate (TPA) and calcium ionophore A23187 in endometrial carcinoma cells. *Carcinogenesis.* 2006;27(3):483-90.

19.	Zhou Q, Xu J, Xu Y, Sun S, and Chen J. Role of ICAM1 in tumor immunity and prognosis of triple-negative breast cancer. *Front Immunol.* 2023;14:1176647.

20.	Tsai ST, Wang PJ, Liou NJ, Lin PS, Chen CH, and Chang WC. ICAM1 Is a Potential Cancer Stem Cell Marker of Esophageal Squamous Cell Carcinoma. *PLoS One.* 2015;10(11):e0142834.

21.	Velastegui E, Vera E, Vanden Berghe W, Munoz MS, and Orellana-Manzano A. "HLA-C: evolution, epigenetics, and pathological implications in the major histocompatibility complex". *Front Genet.* 2023;14:1206034.

22.	Michelakos T, Kontos F, Kurokawa T, Cai L, Sadagopan A, Krijgsman D, et al. Differential role of HLA-A and HLA-B, C expression levels as prognostic markers in colon and rectal cancer. *J Immunother Cancer.* 2022;10(3).

23.	Wang Y, Bi X, Luo Z, Wang H, Ismtula D, and Guo C. Gelsolin: A comprehensive pan-cancer analysis of potential prognosis, diagnostic, and immune biomarkers. *Front Genet.* 2023;14:1093163.

24.	Wang Z, Wang K, Gao X, Liu Z, and Xing Z. Comprehensive analysis of the importance of PLAUR in the progression and immune microenvironment of renal clear cell carcinoma. *PLoS One.* 2022;17(6):e0269595.

25.	Lin L, Zheng X, Qiu C, Dongol S, Lv Q, Jiang J, et al. SIRT1 promotes endometrial tumor growth by targeting SREBP1 and lipogenesis. *Oncol Rep.* 2014;32(6):2831-5.

26.	Korbecki J, Bosiacki M, Barczak K, Lagocka R, Brodowska A, Chlubek D, et al. Involvement in Tumorigenesis and Clinical Significance of CXCL1 in Reproductive Cancers: Breast Cancer, Cervical Cancer, Endometrial Cancer, Ovarian Cancer and Prostate Cancer. *Int J Mol Sci.* 2023;24(8).

27.	Wallace AE, Sales KJ, Catalano RD, Anderson RA, Williams AR, Wilson MR, et al. Prostaglandin F2alpha-F-prostanoid receptor signaling promotes neutrophil chemotaxis via chemokine (C-X-C motif) ligand 1 in endometrial adenocarcinoma. *Cancer Res.* 2009;69(14):5726-33.

28.	Patel S, and Jin L. TMEM173 variants and potential importance to human biology and disease. *Genes Immun.* 2019;20(1):82-9.

29.	Cai Y, Chen X, Lu T, Yu Z, Hu S, Liu J, et al. Single-cell transcriptome analysis profiles the expression features of TMEM173 in BM cells of high-risk B-cell acute lymphoblastic leukemia. *BMC Cancer.* 2023;23(1):372.

30.	Lawal B, Tseng SH, Olugbodi JO, Iamsaard S, Ilesanmi OB, Mahmoud MH, et al. Pan-Cancer Analysis of Immune Complement Signature C3/C5/C3AR1/C5AR1 in Association with Tumor Immune Evasion and Therapy Resistance. *Cancers (Basel).* 2021;13(16).

31.	Sales KJ, Maldonado-Perez D, Grant V, Catalano RD, Wilson MR, Brown P, et al. Prostaglandin F(2alpha)-F-prostanoid receptor regulates CXCL8 expression in endometrial adenocarcinoma cells via the calcium-calcineurin-NFAT pathway. *Biochim Biophys Acta.* 2009;1793(12):1917-28.

32.	Xu F, Xu Y, Xiong JH, Zhang JH, Wu J, Luo J, et al. AOC1 Contributes to Tumor Progression by Promoting the AKT and EMT Pathways in Gastric Cancer. *Cancer Manag Res.* 2020;12:1789-98.

33.	Song F, Dai Q, Grimm MO, and Steinbach D. The Antithetic Roles of IQGAP2 and IQGAP3 in Cancers. *Cancers (Basel).* 2023;15(4).

34.	Wang J, Yang L, Liang F, Chen Y, and Yang G. Integrin alpha x stimulates cancer angiogenesis through PI3K/Akt signaling-mediated VEGFR2/VEGF-A overexpression in blood vessel endothelial cells. *J Cell Biochem.* 2019;120(2):1807-18.

35.	Dillon LM, and Miller TW. The PREX1/Rac signaling axis: Potential as a biomarker and therapeutic target in breast cancer. *Mol Cell Oncol.* 2015;2(3):e996016.

36.	Wan SC, Wu H, Li H, Deng WW, Xiao Y, Wu CC, et al. Overexpression of PREX1 in oral squamous cell carcinoma indicates poor prognosis. *J Mol Histol.* 2020;51(5):531-40.
